# Structural Optimization of Isoquinoline Derivatives from Lycobetaine and Their Inhibitory Activity against Neuroendocrine Prostate Cancer Cells

**DOI:** 10.3390/molecules29184503

**Published:** 2024-09-23

**Authors:** Zhuo Zhang, Qianqian Shen, Yiyi Ji, Yanjie Ma, Haiyang Hou, Huajie Yang, Yinjie Zhu, Yi Chen, Youhong Hu

**Affiliations:** 1 School of Chinese Materia Medica, College of Pharmacy, Nanjing University of Chinese Medicine, No. 138 Xianlin Road, Nanjing 210023, China; s18-zhangzhuo@simm.ac.cn; 2 State Key Laboratory of Chemical Biology, Shanghai Institute of Materia Medica, Chinese Academy of Sciences, Shanghai 201203, China; shenqianqian@simm.ac.cn (Q.S.); houhaiyang@simm.ac.cn (H.H.); ychen@simm.ac.cn (Y.C.); 3 Department of Urology, Renji Hospital, Shanghai Jiao Tong University School of Medicine, Shanghai 200127, China; jiyiyi@sjtu.edu.cn; 4 State Key Laboratory of Drug Research, Shanghai Institute of Materia Medica, Chinese Academy of Sciences, 555 Zu-ChongZhi Road, Shanghai 201203, China; myj1007@simm.ac.cn (Y.M.); yanghuajie@simm.ac.cn (H.Y.); 5 Shandong Laboratory of Yantai Drug Discovery, Bohai Rim Advanced Research Institute for Drug Discovery, Yantai 264117, China; 6 University of Chinese Academy of Sciences, No. 19A Yuquan Road, Beijing 100049, China; 7 School of Pharmaceutical Science and Technology, Hangzhou Institute for Advanced Study, University of Chinese Academy of Sciences, 1st Xiangshan Branch Alley, Hangzhou 310024, China

**Keywords:** NEPC, natural product, natural product derivatives, lycobetaine

## Abstract

Neuroendocrine prostate cancer (NEPC) is a highly aggressive cancer that is resistant to hormone therapy and characterized by poor prognosis, as well as limited therapeutic options. Since the natural product lycobetaine was reported to exhibit good antitumor activities against various types of cancers, we initially simplified the scaffold of lycobetaine to obtain the active compound **1**, an isoquinoline derivative with an aryl moiety substitution at the 4-position, which showed apparent antiproliferative activities against NPEC cell line LASCPC-01 in vitro. Subsequently, we carried out structural optimization and systematic structure–activity relationship (SAR) studies on compound **1**, leading to the discovery of compound **46**, which demonstrated potent inhibitory activities against the LASCPC-01 cell line with an IC_50_ value of 0.47 μM. Moreover, compound **46** displayed remarkable selectivity over prostate cancer cell line PC-3 with a selectivity index greater than 190-fold. Further cell-based mechanism studies revealed that compound **46** and lycobetaine can effectively induce G1 cell cycle arrest and apoptosis dose dependently. However, lycobetaine inhibited the expression of neuroendocrine markers, while compound **46** slightly upregulated these proteins. This suggested that compound **46** might exert its antitumor activities through a different mechanism than lycobetaine, warranting further study.

## 1. Introduction

Prostate cancer (PC) is a global public health concern and the most common non-skin malignancy diagnosed in men in the United States. It ranks second as the leading cause of cancer-related mortality [1,2]. The standard first-line treatment approach for PC is androgen deprivation therapy (ADT), as it is primarily a hormone-driven disease overexpressing both prostate-specific antigen (PSA) and androgen receptor (AR) proteins [3]. Although patients initially respond to this therapy, some who undergo long-term treatment with ADT exhibit castration-resistant prostate cancer (CRPC), and some eventually display the neuroendocrine phenotype, defined as neuroendocrine prostate cancer (NEPC). NEPC is characterized by the expression of neuroendocrine markers such as synaptophysin (SYP), chromogranin A (CHGA), and neural cell adhesion molecule 1 (CD56), and the absence of AR and PSA secretion [4,5]. NEPC is an aggressive variant of prostate cancer that is resistant to AR-targeted therapies [6,7,8], leading to poor prognosis and a median survival of less than one year after diagnosis [9]. Currently, the treatments for this condition focus on platinum-based cures but are not durable and usually have high toxicity [10]. Additionally, several other emerging therapies are undergoing development. For example, various immunotherapy drugs, such as immune checkpoint inhibitors, have been trialed clinically but showed limited efficacy [11,12]. Kinase inhibitors are a second example, such as aurora kinase A inhibitor alisertib, which was in phase II trial but did not meet its primary endpoint [13]. Finally, epigenetic-targeting drugs are represented by EZH2 inhibitors due to EZH2 being overexpressed in NEPC frequently and having been confirmed as a master regulator of NE differentiation of PC cells [14,15,16,17,18,19]. Despite some of these upcoming treatments having achieved partial results in preclinical or clinical research, they are still far from practical application for alleviating patients’ conditions. So, there is still an urgent need for an effective therapy for this disease [20].

**Figure 1:**
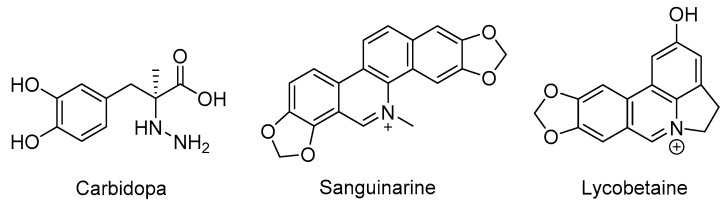
Structures of carbidopa, sanguinarine, and lycobetaine.

As reported, the DOPA decarboxylase (DDC) inhibitor carbidopa has exhibited anti-prostate cancer activity in vitro and in vivo [21,22]. Additionally, DDC is considered as a well-established neuroendocrine differentiation (NED) marker for prostate cancer cells [23,24], which may be crucial in NEPC occurrence [5]. Bearing this in mind, we selected the known DDC inhibitor carbidopa and two other natural products (sanguinarine, which exhibited significant activity against DDC [25], and lycobetaine, which is similar in structure to sanguinarine, are shown in Figure 1) for evaluation of antiproliferative activities against NPEC cell line LASCPC-01 and prostate cancer cell line PC-3. We found that lycobetaine exhibited a more favorable inhibitory activity profile against the LASCPC-01 cell line.

**Figure 2:**
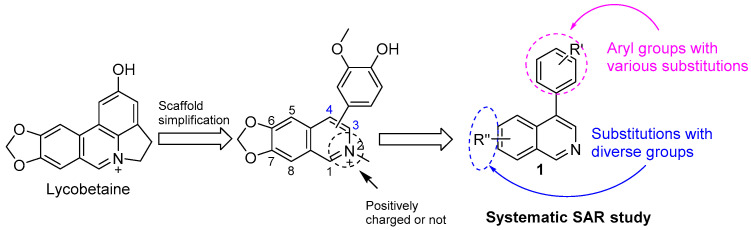
Design strategy of isoquinoline derivatives.

Lycobetaine is a natural inhibitor of mammalian and bacterial type I and type II DNA topoisomerases [26,27,28], previously used clinically to treat gastric and ovarian cancers, though its use was discontinued due to local irritation [29]. This compound can be isolated from amaryllis plants or synthesized by oxidizing lycorine with selenium dioxide [30,31]. In our study, we initially designed and synthesized 3- and 4-substituted isoquinolines to simplify this scaffold (Figure 2) for evaluation of the antiproliferative activities against the NPEC cell line directly, which yielded the active compound **1**. Next, we carried out a systematic SAR study and found compound **46** exhibited the most potent activity against the NEPC cell line in vitro, as well as superb selectivity over the PC-3 prostate cancer cell line.

## 2. Results and Discussion

### 2.1. Chemistry

**Scheme 1:**
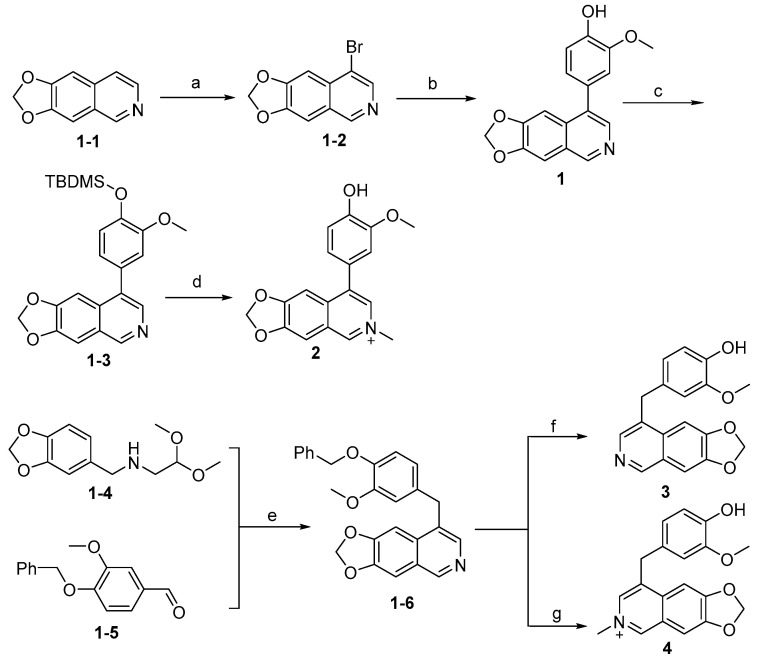
Synthesis of compounds **1**–**4** *^a^*. *^a^* Reagents and conditions: (a) NBS, CCl_4,_ reflux, 12 h, 45%; (b) (4-hydroxy-3-methoxyphenyl)boronic acid, Pd(dppf)Cl_2_, K_2_CO_3_, 1,4-dioxane, H_2_O, N_2_, 85 °C, 12 h, 75%; (c) TBDMSCl, imidazole, DMF, rt, 90%; (d) i: CH_3_I, MeCN, 85 °C, 3 h, sealed tube; ii; TFA, DCM, rt, 3 h, 25%; (e) EtOH, Conc. HCl (37 wt% in water), 100 °C, 30 min, 30%; (f) H_2_, Pd/C, MeOH, 25 °C, 12 h, 85%; (g) i: CH_3_I, MeCN, 85 °C, 3 h, sealed tube; ii: TFA, DCM, rt, 12 h, 20%.

Synthesis of compounds **1**–**4** is outlined in Scheme 1. The intermediate (**1**-**1**) was generated as previously reported [32] and underwent bromination via NBS and then Suzuki coupling reaction in the presence of Pd(dppf)Cl_2_ to give the compound **1**. Compound **1** was further protected by a trimethylsilyl group to give (**1**-**3**), which reacted with methyl iodide, followed by deprotection using the acidic condition to afford compound **2** in moderate yields. The intermediate (**1**-**6**) was prepared from (**1**-**4**) and (**1**-**5**) under strong acidic conditions under high temperature. Intermediate (**1**-**6**) was deprotected by hydrogenation catalyzed by Pd/C to yield **3**, or it reacted with methyl iodide followed by deprotection to give **4**. The intermediates (**1**-**4**) or (**1**-**5**) were synthesized as described in acceptable yields [32,33].

**Scheme 2:**
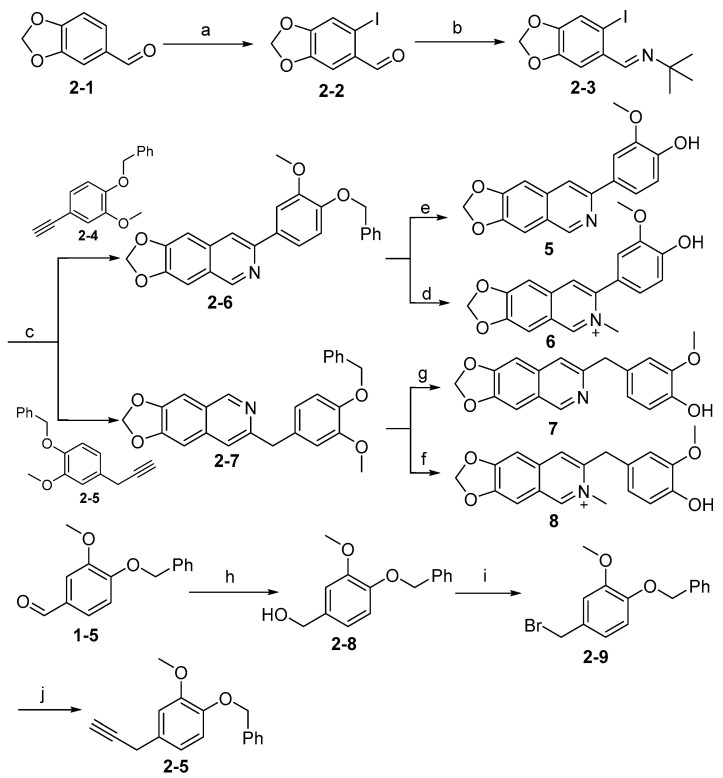
Synthesis of compounds **5**–**8** *^a^*. *^a^* Reagents and conditions: (a) iodine, AgCO_3_, MeOH (dry), N_2_, rt, 12 h, 75%; (b) *tert*-Butylamine, H_2_O, N_2_, rt, 63%; (c) i: PdCl_2_(PPh_3_)_2_, CuI, TEA (dry), N_2_, 55 °C, 6 h; ii: CuI, DMF, N_2_, 100 °C, 12 h, 50~62%; (d) i: CH_3_I, MeCN, 85 °C, 3 h, sealed tube; ii: TFA, DCM, rt, 12 h, 25%; (e) H_2_, Pd/C, MeOH, 25 °C, 12 h, 83%; (f) i: CH_3_I, MeCN, 85 °C, 3 h, sealed tube; ii: TFA, DCM, rt, 12 h, 27%; (g) H_2_, Pd/C, MeOH, 25 °C, 12 h, 75%; (h) NaBH_4_, MeOH, 0 °C–rt, 6 h, 94%; (i) PBr_3_, THF (dry), N_2_, 0 °C, 0.5 h, 30%; (j) Ethynyl magnesium bromide, CuCl, THF, rt–reflux, 21 h, 33%.

Synthesis of target compounds **5**–**8** is shown in Scheme 2. The intermediate (**2**-**3**) was readily accessed from commercially available material (**2**-**1**) via iodination followed by condensation with *tert*-butylamine. Subsequently, intermediate (**2**-**3**) reacted with alkynyl derivatives (**2**-**4**) or (**2**-**5**) to give the intermediates (**2**-**6**) and (**2**-**7**), respectively, via Sonogashira cross-coupling reaction. The intermediates (**2**-**6**) and (**2**-**7**) were deprotected by hydrogenation catalyzed by Pd/C to give **5** and **7**. Alternatively, (**2**-**6**) and (**2**-**7**) were methylated, followed by deprotection using acid to give **6** and **8**, respectively. Intermediate (**2**-**4**) was prepared easily as previously described [34]. Intermediate (**2**-**5**) was synthesized from starting material (**1**-**5**) in three steps in acceptable yields.

**Scheme 3:**
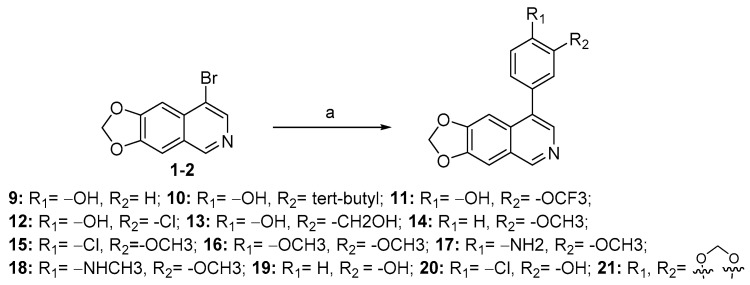
Synthesis of compounds **9**–**21** *^a^*. *^a^* Reagents and conditions: (a) various substituted arylboronic acids or arylboronates, Pd(dppf)Cl_2_, K_2_CO_3_, 1,4-dioxane, H_2_O, N_2_, 85 °C, 12 h, 50–75%.

As depicted in Scheme 3, compounds **9**–**21** were obtained from the key intermediate (**1**-**2**), which was prepared, as illustrated in Scheme 1, through Suzuki-coupling reaction with various substituted arylboronic acids or arylboronates, resulting in moderate to high yields.

**Scheme 4:**
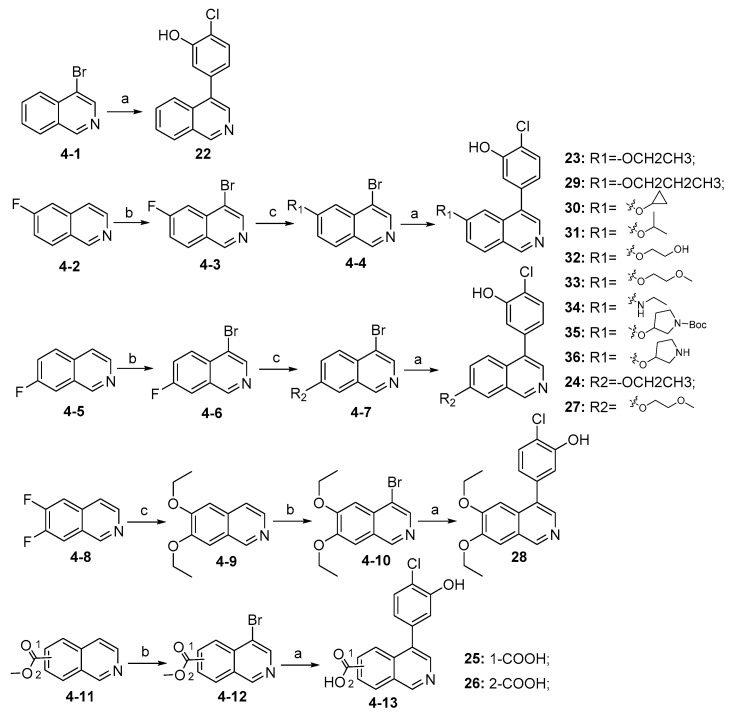
Synthesis of compounds **22**–**36** *^a^*. *^a^* Reagents and conditions: (a) (4-chloro-3-hydroxyphenyl)boronic acid, Pd(dppf)Cl_2_, K_2_CO_3_, 1,4-dioxane, H_2_O, N_2_, 85 °C, 12 h, 45–85%; (b) NBS, acetic acid, 90 °C, 12 h, 25~50%; (c) various substituted alcohols or amines, NaH, DMF, 0 °C–rt, 10 h, 40–85%.

Synthesis of compounds **22**–**36** is described in Scheme 4. Compound **22** was directly generated from commercially available material (**4**-**1**) via Suzuki coupling reaction. Starting materials (**4**-**2**) and (**4**-**5**) were brominated using NBS to afford (**4**-**3**) and (**4**-**6**), respectively, which followed by nucleophilic substitution reactions to give intermediates (**4**-**4**) and (**4**-**7**). Intermediates (**4**-**4**) and (**4**-**7**) further coupled with (4-chloro-3-hydroxyphenyl) boronic acid catalyzed by Pd(dppf)Cl_2_ to produce compounds **23**–**24**, **27**, **29**–**36**. Compound **28** was synthesized from commercially available material (**4**-**8**) via nucleophilic substitution reaction, followed by bromination and Suzuki coupling reaction. Compounds **25** and **26** were prepared from commercially available (**4**-**11**) through bromination with NBS followed by Suzuki coupling reaction, during which the process part of the methyl carboxylates were hydrolyzed to carboxylic acids.

**Scheme 5:**
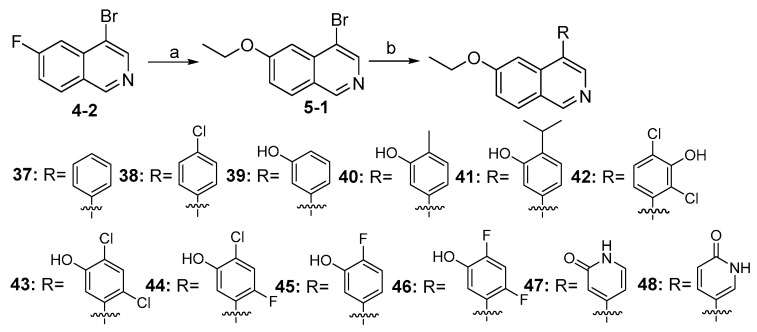
Synthesis of compounds **37**–**48** *^a^*. *^a^* Reagents and conditions: (a) NaOEt (20 wt% in EtOH), 0 °C–rt, 12 h, 45%; (b) various substituted arylboronic acids or arylboronates, Pd(dppf)Cl_2_, K_2_CO_3_, 1,4-dioxane, H_2_O, N_2_, 85 °C, 12 h, 60–86%.

As shown in Scheme 5, compounds **37**–**48** were synthesized from the intermediate (**4**-**2**) with similar procedures, as depicted in Scheme 4.

### 2.2. Discovery of the Lead Compound and SAR Study

#### 2.2.1. Lycobetaine Exhibited Superior Antiproliferative Activity against LASCPC-01 Cell Line and Showed Greater Selectivity over PC-3

Initially, we selected the known DDC inhibitor carbidopa, along with the natural products sanguinarine and lycobetaine, to evaluate their antiproliferative activity against the NEPC cell line LASCPC-01 and the prostate cancer cell line PC-3. The results, shown in Table 1, indicated that both natural products exhibited significant activity against the LASCPC-01 and PC-3 cell lines. Furthermore, lycobetaine demonstrated greater activity compared to sanguinarine and exhibited excellent selectivity over the PC-3 cell line.

#### 2.2.2. Identification of 4-Aryl-Substituted Isoquinoline Derivative **1** as the Active Compound

Based on the significant antiproliferative activity of lycobetaine against the LASCPC-01 cell line, as well as its apparent selectivity over the PC-3 cell line, we embarked on structural dissection and simplification of lycobetaine. As indicated in Figure 2, we designed and synthesized several compounds with the aryl moiety substituted at the 3- or 4-positions on the [1,3]dioxolo[4,5-g]isoquinoline core and evaluated their proliferation inhibitory activities against the NEPC cell line LASCPC-01. At the same time, we investigated the effects of bearing a positive charge on the activities. The results are shown in Table 2; the 3-substituted compounds exhibited poor activities (**5**–**7**) except for compound **8**, which bears a positive charge and has a carbon atom between the aryl moiety and the core and displayed moderate proliferation inhibitory activity against the LASCPC-01 cell line. The 4-substituted compounds lost activity when they carried a positive charge (**2** and **4**). However, without a positive charge, the 4-substituted compound **1** showed better activity than **3** and performed the best among these compounds; therefore, we selected **1** for further optimization.

#### 2.2.3. SAR Exploration at the 4-Substitution of Isoquinoline

Subsequently, we carried out the SAR study on the phenyl group moiety at the 4-position of isoquinoline to determine the antiproliferative activities against the LASCPC-01 cell line. The results, as shown in Table 3, indicated that compound **9** lost its activity when only the para-position hydroxyl group was substituted. When the OCH_3_ group in compound **1** was replaced by *tert*-butyl, OCF_3_, Cl, or CH_2_OH, the activities were reduced (**10**–**13**). When the hydroxyl group of compound **1** was removed, the activity was significantly decreased (**14**). Based on the structure of compound **14**, when Cl (**15**), methoxy (**16**), NH_2_ (**17**), or NHCH_3_ (**18**) were introduced to the para-position, the activities were also undesirable. To our delight, the meta-position hydroxyl group-substituted compounds **19** and **20** showed significant activities comparable to the compound **1**. These results indicated that the presence of a hydroxyl group at the meta-position may be crucial for the activities of these analogs. Compound **21**, which was cyclized between para and meta positions, also lost activity.

#### 2.2.4. SAR Exploration at the 6- and 7-Positions of Isoquinoline

Due to the facile glucuronidation metabolism of the phenolic hydroxyl group, we substituted the 4-position of isoquinoline with a 3-hydroxy-4-chlorophenyl group, considering that the introduction of a chlorine atom close to the hydroxyl group might reduce glucuronidation metabolism due to steric and electronic effects. We then performed structural optimizations at the 6- and 7-positions. The results are shown in Table 4. Compared with compound **20**, the removal of substitutions at sites 6 and 7 yielded compound **22**, which exhibited decreased activity. Compound **23**, which introduced an ethoxy group at the 6-position, displayed excellent activity, better than that of compound **20**. However, when the ethoxy group was moved to the 7-position (**24**), the activity was lost. When both the 6- and 7-positions were substituted with ethoxy groups to afford compound **28**, the activity was comparable to that of **20**. Replacing the ethoxy group with a propoxy group (**29**), isopropoxy group (**31**), or cyclopropoxy group (**30**) at the 6-position significantly reduced the activity of the compounds, suggesting an intolerance to large steric moieties at this position. When the 6- or 7-position was substituted by a carboxyl group (**25**–**26**), the activities of the compounds decreased significantly, with compound **25** losing the activity. This suggests that the 6- or 7-position of the isoquinoline core cannot bear the electron-withdrawing groups while maintaining activity. When the 6-position was substituted with an ethyl-amino group or an ethylene glycol moiety, yielding compounds **34** and **32**, respectively, both compounds showed good activities, similar to that of compound **23**. In addition, when the 6-position was substituted with a large pyrrolidin-3-yloxy group or a Boc-protected pyrrolidin-3-yloxy group (**35** and **36**), the activities of these compounds decreased compared to compound **23**. These results indicated that simplified, less bulky electron-donating groups at the 6-position could enhance the antiproliferative activity against the LASCPC-01 cell line.

#### 2.2.5. Further Investigation at the 4-Substitution of Isoquinoline Leads to Discovery

In light of the remarkable activity exhibited by compound **23**, we further performed a more refined SAR study at the 4-substitution of isoquinoline with a 6-position ethoxy group substituted. The results are shown in Table 5. Removal of both the hydroxyl group and chlorine from the phenyl moiety, or removal of the hydroxyl group from the meta-position only, resulted in the synthesis of compounds **37** and **38**, which exhibited reduced activity. In contrast, when retaining the hydroxyl group at the meta-position only, the activity of **39** was increased. These results confirmed the crucial role of the hydroxy group substitution at the meta-position for the activity and the necessity of modifying the para-position of the phenyl ring with diverse substitutions. Fixing the meta-position with a hydroxyl group substituted, the compounds **40** and **41** with electron-donating or bulky substitutions at the para-position would decrease the activity. In order to block the metabolic soft spot at the para-position of the hydroxyl group, the different chlorine atoms were introduced based on the structure of **23** to design and synthesize the compounds **42**–**43**. Apparently, compound **43** with another chloride at the para-position of the hydroxyl group maintained its activity. With replacement of the chlorine with fluorine at the para-position of the hydroxyl group, the activity of **44** was reduced. However, when introducing F instead of Cl, the activities of compounds **45**–**46** were enhanced significantly and compound **46** demonstrated an IC_50_ value of 0.47 μM against the LASCPC-01 cell line. When the phenyl substituents at the 4-position of isoquinoline were replaced by pyridone moieties with the potential hydroxyl group, the activities of these compounds (**47**–**48**) decreased.

#### 2.2.6. The Investigation of the Representative Compounds for Selectivity

Based on the significant antiproliferative activities exhibited by certain compounds against the NEPC LASCPC-01 cell line, we further selected compounds that performed well to determine their antiproliferative inhibitory activities against the prostate cancer PC-3 cell line (shown in Table 6). Most of these compounds displayed outstanding selectivity toward the LASCPC-01 cell line over PC-3, especially compound **46**, which showed greater than 190-fold for selectivity and had a low IC_50_ value of 0.47 μM against the NEPC LASCPC-01 cell line.

### 2.3. Compound ***46*** Apparently Induced G1 Cell Cycle Arrest and Apoptosis

**Figure 3:**
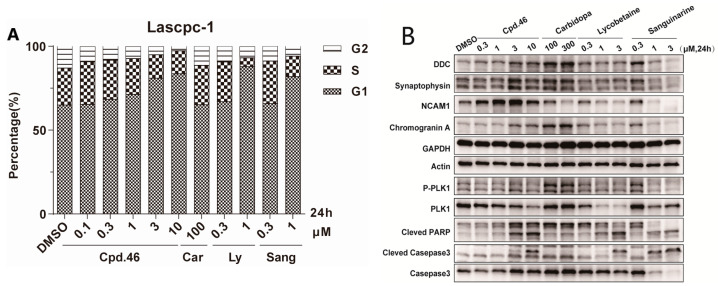
Cell-based mechanism study of compound **46** (Cpd.**46**), carbidopa (Car), lycobetaine (Ly), and sanguinarine (Sang) in LASCPC-01 cell line. (**A**) Cells treated with DMSO or indicated concentrations of compounds for 24 h were analyzed for DNA content by flow cytometry. (**B**) Western blot showing the expression of neuroendocrine-related proteins and apoptosis markers (cleaved-PARP, cleved-casepase3, and casepase3) in LASCPC-01 cell line treated with different concentrations of compounds.

To determine whether inhibition of cell proliferation was associated with cell cycle arrest, LASCPC-01 cells were treated with DMSO or various concentrations of compound **46**, and the relative DNA content of cells was then analyzed by flow cytometry. The results, as indicated in Figure 3A, show that compound **46** effectively induced G1 cell cycle arrest dose dependently, which was comparable to natural products lycobetaine and sanguinarine, while carbidopa showed no activity. Further, we investigated the impact of these compounds on the expression of related proteins. The results, as shown in Figure 3B, indicate that natural products lycobetaine and sanguinarine significantly reduced the expression of neuroendocrine markers DDC, SYN, NCAM1, and CHGA. However, compound **46** did not exhibit an inhibitory effect on the expression of these neuroendocrine markers. Instead, it showed a slightly dose-dependent upregulation trend, which is contrary to our expectations. In addition, we also observed that compound **46** and the two natural compounds apparently induced cell apoptosis dose dependently, manifested by the upregulation of cleaved-PARP and cleaved-casepase3 (Figure 3B). Additionally, DDC inhibitor carbidopa showed only weak activities at high concentrations. Lycobetaine is a known DNA topoisomerase inhibitor [35] and sanguinarine exhibits antitumor activity through its effects on various signaling pathways, including protein kinases and NF-κB [36]. And here, we also found that two compounds decreased the tested neuroendocrine-related proteins in LASCPC-01 cells, which might contribute to their antiproliferative activity against the NPEC cell line. Although compound **46** exhibited selective inhibition on the proliferation of NPEC cells, like the other two natural products, it had no effect on the level of neuroendocrine-related proteins, suggesting that other mechanisms exist and need to be further studied.

## 3. Experimental Section

### 3.1. General Information

All commercially available reagents were purchased and used without further purification unless otherwise indicated. All reactions sensitive to air were performed under a nitrogen (N_2_) atmosphere. All reactions were monitored by thin-layer chromatography (TLC) on precoated TLC glass plates (silica gel GF254, 0.2 ± 0.03 mm thickness). TLC glass plates were developed in a covered chamber and were visualized under ultraviolet light using a 254 nm fluorescent indicator. Flash column chromatography was completed with 200–300 mesh silica gel. NMR spectra were recorded on a Bruker (Billerica, MA, USA) 400 (^1^H: 400 MHz, ^13^C NMR: 100 MHz) or Bruker 600 (^1^H: 600 MHz, ^13^C NMR: 150 MHz) spectrometer. Chemical shifts were described in δ (ppm) units relative to an internal standard (TMS). The coupling constants were given in Hz. Purity of representative compounds was performed on an Agilent (Santa Clara, CA, USA) 1200 series LC system (Agilent Chem-Station Rev.B.03.01; column, ZORBAX Eclipse XD B-C18, 4.6 mm × 150 mm, 5 μm; flow rate, 0.8 mL/min; UV wavelength, maximal absorbance at 254 nm; temperature, 25 °C; injection volume, 15 μL; mobile phase, solvent A: H_2_O (with 0.1% HCOOH) and solvent B: MeOH). The gradient elution: 0~3 min, B: 30~30%; 3~10 min, B: 30~70%; 10~12 min, B: 70~70%; 12~16 min, B: 70~90%; 16~22 min, B: 90~90%; 22~24 min, B: 90~30%; 24~28 min, B: 30~30%. Low-resolution mass spectral (LRMS) data were determined on an Agilent 1200 series LC-MS spectrometer and high-resolution mass spectral (HRMS) data on a Micromass Ultra Q-Tof.

### 3.2. Synthesis of Target Compounds

#### 3.2.1. Synthesis of Intermediate (**1**-**2**)

Intermediate (**1**-**1**) was synthesized as described [32]. To a solution of (**1**-**1**) (300 mg, 1.73 mmol) in CCl_4_ (10 mL) was added NBS (617 mg, 3.47 mmol), and the reaction mixture was refluxed at 85 °C for 12 h. Upon completion of the reaction, the volatiles were removed in vacuo. The resulting mixture was then diluted with EtOAc (20 mL) and water (10 mL). The organic layer was separated and washed with brine (10 mL × 2), dried over Na_2_SO_4_, filtrated, and evaporated under reduced pressure. The residue was purified by flash chromatography on silica gel (10–20% EA in PE) to give (**1**-**2**) as a white solid (217 mg, 45%). ^1^H NMR (400 MHz, Chloroform-d) δ 8.89 (s, 1H), 8.56 (s, 1H), 7.46 (s, 1H), 7.19 (s, 1H), 6.16 (s, 1H). ESI-MS: *m*/*z* = 252.0 [M + H]^+^.

#### 3.2.2. 4-([1,3]Dioxolo[4,5-g]isoquinolin-8-yl)-2-methoxyphenol (**1**)

Method A. To a solution of (**1**-**2**) (100 mg, 0.40 mmol) in 1,4-dioxane/H_2_O (10/1 mL) at room temperature under N_2_ atmosphere were added (4-hydroxy-3-methoxyphenyl) boronic acid (81 mg, 0.48 mmol), Pd(dppf)Cl_2_ (30 mg, 0.04 mmol), K_2_CO_3_ (110 mg, 0.8 mmol). After stirring at 85 °C for 12 h, the mixture was cooled to room temperature, and the volatiles were removed in vacuo. The resulting mixture was then diluted with EtOAc (20 mL) and water (10 mL). The organic layer was separated and washed with brine (10 mL × 2), dried over Na_2_SO_4_, filtrated, and evaporated under reduced pressure. The residue was purified by flash chromatography on silica gel (0–5% MeOH in DCM) to give **1** as a white solid (89 mg, 75%). M.p. 214–216 °C ^1^H NMR (500 MHz, Chloroform-d) δ 8.99 (s, 1H), 8.23 (s, 1H), 8.17 (s, 1H), 7.55 (s, 1H), 7.14 (s, 1H), 7.02 (d, *J* = 2.0 Hz, 1H), 6.94 (d, *J* = 8.0 Hz, 1H), 6.88 (dd, *J* = 8.0, 2.0 Hz, 1H), 6.20 (s, 2H), 3.81 (s, 3H). ^13^C NMR (126 MHz, DMSO) δ 151.1, 149.0, 148.0, 147.7, 146.5, 141.8, 132.4, 131.8, 127.7, 125.6, 122.2, 115.7, 113.7, 103.2, 102.0, 100.1, 55.7. HRMS (ESI+) calcd for C_17_H_14_NO_4_+ (M + H^+^): 296.0917, found: 296.0919. HPLC analysis: 9.706 min, 99.6% purity.

#### 3.2.3. Synthesis of Intermediate (**1**-**3**)

To a solution of **1** (100 mg, 0.34 mmol) in dry DMF (5 mL) at 0 °C under N_2_ atmosphere were added imidazole (46 mg, 0.68 mmol) and TBDMSCl (62 mg, 0.41 mmol), and then the mixture was stirred at room temperature for 12 h. Upon completion of the reaction, the resulting mixture was then diluted with EtOAc (20 mL) and water (10 mL). The organic layer was separated and washed with brine (10 mL × 2), dried over Na_2_SO_4_, filtrated, and evaporated under reduced pressure. The residue was purified by flash chromatography on silica gel (0–15% EA in PE) to give (**1**-**3**) as a white solid (125 mg, 90%). ^1^H NMR (400 MHz, Chloroform-d) δ 8.95 (s, 1H), 8.32 (s, 1H), 7.24 (s, 1H), 7.19 (s, 1H), 6.97 (d, *J* = 7.9 Hz, 1H), 6.95–6.87 (m, 2H), 6.08 (s, 2H), 3.83 (s, 3H), 1.04 (s, 9H), 0.23 (s, 6H). ESI-MS: *m*/*z* = 410.2 [M + H]^+^.

#### 3.2.4. 8-(4-Hydroxy-3-methoxyphenyl)-6-methyl-[1,3]dioxolo[4,5-g]isoquinolin-6-ium (**2**)

Method B. To a sealed tube containing a solution of (**1**-**3**) (125 mg, 0.31 mmol) in acetonitrile (5 mL) was added methyl iodide (66 mg, 0.47 mmol) and the mixture was stirred at 85 °C for 3 h. Subsequently, the reaction mixture was cooled to room temperature followed by evaporation of the volatiles under vacuum. The resulting mixture was dissolved in DCM/TFA (10/5 mL) at room temperature and stirred for 3 h, after which the volatiles were removed in vacuo again. The residue was purified by flash chromatography on silica gel (0–5% MeOH in DCM) to give **2** as a white solid (24 mg, 25%). ^1^H NMR (600 MHz, DMSO-d_6_) δ 9.48 (s, 1H), 8.53 (s, 1H), 8.42 (s, 1H), 7.83 (s, 1H), 7.41 (s, 1H), 7.10 (d, *J* = 2.0 Hz, 1H), 7.04 (d, *J* = 8.0 Hz, 1H), 6.97 (dd, *J* = 8.0, 2.0 Hz, 1H), 6.42 (s, 2H), 4.38 (s, 3H), 3.83 (s, 3H). ESI-MS: *m*/*z* = 311.1 [M + H]^+^.

#### 3.2.5. Synthesis of Intermediate (**1**-**6**)

To a solution of (**1**-**5**) (1.5 g, 6.27 mmol) in the mixture of EtOH and Conc. HCl (20/20 mL) was added (**1**-**4**) (1.5 g, 6.27 mmol). The reaction mixture was stirred at 100 °C for 3 h. Upon completion of the reaction, NaHCO_3_ (aq) was added to adjust the pH to 8~9, and then the volatiles were evaporated under vacuum. The resulting mixture was diluted with EtOAc (60 mL) and water (30 mL). The organic layer was separated and washed with brine (30 mL × 2), dried over Na_2_SO_4_, filtrated, and evaporated under reduced pressure. The residue was purified by flash chromatography on silica gel (5–20% EA in PE) to give (**1**-**6**) as a yellow solid (752 mg, 30%). ^1^H NMR (400 MHz, DMSO-d_6_) δ 8.89 (s, 1H), 8.14 (s, 1H), 7.88 (s, 1H), 7.47 (s, 1H), 7.29–7.04 (m, 5H), 6.78–6.72 (m, 1H), 6.60 (m, *J* = 21.1 Hz, 2H), 6.18 (s, 2H), 5.14 (s, 2H), 4.13 (s, 2H), 3.52 (s, 3H). ESI-MS: *m*/*z* = 400.2 [M + H]^+^.

#### 3.2.6. 4-([1,3]Dioxolo[4,5-g]isoquinolin-8-ylmethyl)-2-methoxyphenol (**3**)

To a solution of (**1**-**6**) (100 mg, 0.25 mmol) in MeOH (20 mL) was added Pd/C (10 mg). The reaction was stirred under H_2_ atmosphere at room temperature for 12 h. Upon completion of the reaction, the mixture was filtered to remove the solid residue, and the filtrate was evaporated under vacuum. And then, the resulting mixture was diluted with EtOAc (20 mL) and water (10 mL). The organic layer was separated and washed with brine (10 mL × 2), dried over Na_2_SO_4_, filtrated, and evaporated under reduced pressure. The residue was purified by flash chromatography on silica gel (0–5% MeOH in DCM) to give **3** as a white solid (66 mg, 85%). ^1^H NMR (400 MHz, DMSO-d_6_) δ 8.91 (s, 1H), 8.75 (s, 1H), 8.23 (s, 1H), 7.46 (s, 1H), 7.37 (s, 1H), 6.88 (d, *J* = 2.0 Hz, 1H), 6.64 (d, *J* = 8.0 Hz, 1H), 6.55 (dd, *J* = 8.0, 2.0 Hz, 1H), 6.17 (s, 2H), 4.16 (s, 2H), 3.70 (s, 3H). ESI-MS: *m*/*z* = 310.1 [M + H]^+^.

#### 3.2.7. 8-(4-Hydroxy-3-methoxybenzyl)-6-methyl-[1,3]dioxolo[4,5-g]isoquinolin-6-ium (**4**)

Method B. Compound **4** was prepared from the intermediate (**1**-**6**) (100 mg, 0.25 mmol) and methyl iodide (53 mg, 0.38 mmol) in a sealed tube using the method for the synthesis of compound **2**. This afforded a white solid (16 mg, 20%). ^1^H NMR (400 MHz, DMSO-d_6_) δ 9.41 (s, 1H), 8.98 (s, 1H), 8.45 (s, 1H), 7.74 (s, 1H), 7.72 (s, 1H), 6.97 (d, *J* = 1.9 Hz, 1H), 6.71–6.58 (m, 2H), 6.39 (s, 2H), 4.35 (s, 3H), 4.31 (s, 2H), 3.73 (s, 3H). ESI-MS: *m*/*z* = 324.1 [M + H]^+^.

#### 3.2.8. Synthesis of Intermediate (**2**-**2**)

To a solution of commercially available intermediate (**2**-**1**) (1 g, 6.7 mmol) in dry MeOH (30 mL) were added iodine (1.86 g, 7.3 mmol) and AgCO_3_ (1.14 g, 6.7 mmol). The reaction was stirred under N_2_ atmosphere at room temperature for 12 h. Upon completion of the reaction, the volatiles were removed in vacuo. The resulting mixture was diluted with EtOAc (60 mL) and water (30 mL). The organic layer was separated and washed with brine (30 mL × 2), dried over Na_2_SO_4_, filtrated, and evaporated under reduced pressure. The residue was purified by flash chromatography on silica gel (0–20% EA in PE) to give (**2**-**2**) as a white solid (1.39 g, 75%). ^1^H NMR (400 MHz, DMSO-d_6_) δ 9.79 (s, 1H), 7.60 (s, 1H), 7.26 (s, 1H), 6.18 (s, 2H). ESI-MS: *m*/*z* = 276.9 [M + H]^+^.

#### 3.2.9. Synthesis of Intermediate (**2**-**3**)

To a solution of intermediate (**2**-**2**) (1.39 g, 5.0 mmol) in water (30 mL) was added *tert*-butylamine (1.1 g, 15 mmol). The reaction was stirred under N_2_ atmosphere at room temperature for 12 h. Upon completion of the reaction, the volatiles were removed in vacuo. The resulting mixture was diluted with EtOAc (60 mL) and water (30 mL). The organic layer was separated and washed with brine (30 mL × 2), dried over Na_2_SO_4_, filtrated, and evaporated under reduced pressure to afford (**2**-**3**) as a white solid without further purification. (1.04 g, 63%). ^1^H NMR (400 MHz, DMSO-d_6_) δ 8.27 (s, 1H), 7.45 (s, 1H), 7.35 (s, 1H), 6.10 (s, 2H), 1.23 (s, 9H). ESI-MS: *m*/*z* = 332.0 [M + H]^+^.

#### 3.2.10. Synthesis of Intermediate (**2**-**5**)

To a solution of CuCl (16 mg, 0.15 mmol) in dry THF (5 mL) was added ethynyl magnesium bromide (126 mg, 0.98 mmol). The reaction mixture was stirred at room temperature for 10 min; intermediate (**2**-**9**) (150 mg, 0.49 mmol) was added and refluxed at 75 °C for 21 h. Upon completion of the reaction, the mixture was quenched by addition of 5 mL NH_4_Cl (aq), followed by dilution with EtOAc (20 mL) and water (5 mL). The organic layer was separated and washed with brine (10 mL × 2), dried over Na_2_SO_4_, filtrated, and evaporated under reduced pressure. The residue was purified by flash chromatography on silica gel (0–5% MeOH in DCM) to give (**2**-**5**) as a yellow solid (41 mg, 33%). ^1^H NMR (400 MHz, Chloroform-d) δ 7.48–7.28 (m, 5H), 6.91 (d, *J* = 1.6 Hz, 1H), 6.84–6.79 (m, 2H), 5.14 (s, 2H), 3.90 (s, 3H), 3.55 (d, *J* = 2.7 Hz, 2H), 2.18 (t, *J* = 2.7 Hz, 1H). ESI-MS: *m*/*z* = 253.1 [M + H]^+^.

#### 3.2.11. Synthesis of Intermediate (**2**-**6**)

Intermediate (**2**-**4**) was synthesized as described [34]. Dry Et_3_N (2 mL), PdCl_2_(PPh_3_)_2_ (21 mg, 0.03 mmol), (**2**-**3**) (100 mg, 0.3 mmol), (**2**-**4**) (86 mg, 0.36 mmol), and CuI (3 mg, 0.015 mmol) were placed in a two-neck reaction flask. The reaction was stirred at 55 °C under N_2_ atmosphere for 6 h. After the reaction was completed, the reaction mixture was cooled to room temperature, the precipitates were filtered off and washed with ether, and the filtrate was evaporated under reduced pressure. The residue obtained was transferred to a two-dram vial and DMF (5 mL) and CuI (3 mg, 0.015 mmol) were added. The reaction was stirred at 100 °C under N_2_ atmosphere for 12 h. Upon completion of the reaction, the mixture was diluted with EtOAc (20 mL) and water (10 mL). The organic layer was separated and washed with brine (10 mL × 2), dried over Na_2_SO_4_, filtrated, and evaporated under reduced pressure. The residue was purified by flash chromatography on silica gel (0–5% MeOH in DCM) to afford (**2**-**6**) as a white solid. (58 mg, 50%). ^1^H NMR (400 MHz, Chloroform-d) δ 9.03 (s, 1H), 7.82 (s, 1H), 7.73 (d, *J* = 2.1 Hz, 1H), 7.53–7.45 (m, 6H), 7.20 (s, 1H), 7.10 (s, 1H), 6.98 (d, *J* = 8.3 Hz, 1H), 6.10 (s, 2H), 5.23 (s, 2H), 4.03 (s, 3H). ESI-MS: *m*/*z* = 386.1 [M + H]^+^.

#### 3.2.12. Synthesis of Intermediate (**2**-**7**)

Dry Et_3_N (2 mL), PdCl_2_(PPh_3_)_2_ (21 mg, 0.03 mmol), (**2**-**3**) (100 mg, 0.3 mmol), (**2**-**5**) (91 mg, 0.36 mmol), and CuI (3 mg, 0.015 mmol) were placed in a two-neck reaction flask. The reaction was stirred at 55 °C under N_2_ atmosphere for 6 h. After the reaction was completed, the mixture was cooled to room temperature, the precipitates were filtered off and washed with ether, and the filtrate was evaporated under reduced pressure. The residue obtained was transferred to a two-dram vial and DMF (5 mL) and CuI (3 mg, 0.015 mmol) were added. The reaction was stirred at 100 °C under N_2_ atmosphere for 12 h. Upon completion of the reaction, the mixture was diluted with EtOAc (20 mL) and water (10 mL). The organic layer was separated and washed with brine (10 mL × 2), dried over Na_2_SO_4_, filtrated, and evaporated under reduced pressure. The residue was purified by flash chromatography on silica gel (0–5% MeOH in DCM) to afford (**2**-**7**) as a white solid. (64 mg, 53%). ^1^H NMR (400 MHz, DMSO-d_6_) δ 8.92 (s, 1H), 7.39 (d, *J* = 4.1 Hz, 2H), 7.23–7.10 (m, 7H), 6.75 (s, 1H), 6.61 (s, 1H), 6.16 (s, 2H), 3.98 (s, 2H), 3.82 (s, 2H), 3.73 (s, 3H). ESI-MS: *m*/*z* = 400.1 [M + H]^+^.

#### 3.2.13. Synthesis of Intermediate (**2**-**8**)

To a solution of (**1**-**5**) (1 g, 4.1 mmol) in MeOH (30 mL) at 0 °C was added NaBH_4_ (312 mg, 8.2 mmol). Subsequently, the reaction was recovered to room temperature and stirred for 6 h. Upon completion of the reaction, the solvent was removed under vacuum. The resulting mixture was then diluted with EtOAc (30 mL) and water (15 mL). The organic layer was separated and washed with brine (15 mL × 2), dried over Na_2_SO_4_, filtrated, and evaporated under reduced pressure. The residue was purified by flash chromatography on silica gel (0–5% MeOH in DCM) to give (**2**-**8**) as a white solid (941 mg, 94%). ^1^H NMR (400 MHz, DMSO-d_6_) δ 7.59–7.32 (m, 5H), 7.01–6.45 (m, 4H), 5.07 (s, 2H), 4.41 (s, 2H), 3.77 (s, 3H). ESI-MS: *m*/*z* = 245.1 [M + H]^+^.

#### 3.2.14. Synthesis of Intermediate (**2**-**9**)

To a solution of (**2**-**8**) (941 mg, 3.9 mmol) in dry THF (20 mL) at 0 °C under N_2_ atmosphere, PBr_3_ (2.1 g, 7.8 mmol) was slowly added dropwise. The reaction mixture was stirred at 0 °C for 0.5 h, then quenched by the addition of 5 mL water. The volatiles were removed under vacuum and the resulting mixture was diluted with EtOAc (30 mL) and water (15 mL). The organic layer was separated and washed with brine (15 mL × 2), dried over Na_2_SO_4_, filtrated, and evaporated under reduced pressure. The residue was purified by flash chromatography on silica gel (0–5% EA in PE) to give (**2**-**9**) as a white solid (358 mg, 30%). ^1^H NMR (400 MHz, Chloroform-d) δ 7.47–7.29 (m, 5H), 6.94 (d, *J* = 2.0 Hz, 1H), 6.88 (dd, *J* = 8.2, 2.0 Hz, 1H), 6.82 (d, *J* = 8.2 Hz, 1H), 5.16 (s, 2H), 4.48 (s, 2H), 3.91 (s, 3H). ESI-MS: *m*/*z* = 307.3 [M + H]^+^.

#### 3.2.15. 4-([1,3]Dioxolo[4,5-g]isoquinolin-7-yl)-2-methoxyphenol (**5**)

To a solution of intermediate (**2**-**6**) (58 mg, 0.15 mmol) in MeOH (20 mL) was added Pd/C (10 mg). The reaction was stirred under H_2_ atmosphere at room temperature for 12 h. Upon completion of the reaction, the precipitates were filtered off and washed with MeOH (10 mL), and the filtrate was evaporated under vacuum. The resulting mixture was diluted with EtOAc (20 mL) and water (10 mL). The organic layer was separated and washed with brine (10 mL × 2), dried over Na_2_SO_4_, filtrated, and evaporated under reduced pressure. The residue was purified by flash chromatography on silica gel (0–5% MeOH in DCM) to afford **5** as a white solid. (37 mg, 83%). ^1^H NMR (600 MHz, Chloroform-d) δ 9.03 (s, 1H), 7.84 (s, 1H), 7.73 (d, *J* = 2.0 Hz, 1H), 7.53 (dd, *J* = 8.2, 2.0 Hz, 1H), 7.20 (s, 1H), 7.11 (s, 1H), 7.02 (d, *J* = 8.2 Hz, 1H), 6.11 (s, 2H), 4.03 (s, 3H). ESI-MS: *m*/*z* = 296.1 [M + H]^+^.

#### 3.2.16. 7-(4-Hydroxy-3-methoxyphenyl)-6-methyl-[1,3]dioxolo[4,5-g]isoquinolin-6-ium (**6**)

Method B. Compound **6** was prepared from the intermediate (**2**-**6**) (100 mg, 0.26 mmol) and methyl iodide (54 mg, 0.39 mmol) in a sealed tube using the method for the synthesis of compound **2**. This afforded a white solid (20 mg, 25%). ^1^H NMR (600 MHz, DMSO-d_6_) δ 9.61 (s, 1H), 8.48 (s, 1H), 8.19 (s, 1H), 7.74 (s, 1H), 7.64 (s, 1H), 7.23 (s, 1H), 7.07–6.91 (m, 2H), 6.44 (s, 2H), 4.14 (s, 3H), 3.82 (s, 3H). ESI-MS: *m*/*z* = 310.1 [M + H]^+^.

#### 3.2.17. 4-([1,3]Dioxolo[4,5-g]isoquinolin-7-ylmethyl)-2-methoxyphenol (**7**)

To a solution of intermediate (**2**-**7**) (100 mg, 0.25 mmol) in MeOH (20 mL) was added Pd/C (10 mg). The reaction was stirred under H_2_ atmosphere at room temperature for 12 h. Upon completion of the reaction, the precipitates were filtered off and washed with MeOH (10 mL), and the filtrate was evaporated under vacuum. The resulting mixture was diluted with EtOAc (20 mL) and water (10 mL). The organic layer was separated and washed with brine (10 mL × 2), dried over Na_2_SO_4_, filtrated, and evaporated under reduced pressure. The residue was purified by flash chromatography on silica gel (0–5% MeOH in DCM) to afford **7** as a white solid (58 mg, 75%). ^1^H NMR (400 MHz, DMSO-d_6_) δ 8.93 (s, 1H), 8.74 (s, 1H), 7.41 (s, 2H), 7.25 (s, 1H), 6.86 (d, *J* = 1.7 Hz, 1H), 6.72–6.62 (m, 2H), 6.17 (s, 2H), 4.02 (s, 2H), 3.71 (s, 3H). ESI-MS: *m*/*z* = 310.1 [M + H]^+^.

#### 3.2.18. 7-(4-Hydroxy-3-methoxybenzyl)-6-methyl-[1,3]dioxolo[4,5-g]isoquinolin-6-ium (**8**)

Method B. Compound **8** was prepared from the intermediate (**2**-**7**) (100 mg, 0.25 mmol) and methyl iodide (55 mg, 0.38 mmol) in a sealed tube using the method for the synthesis of compound **2**. This afforded a white solid (22 mg, 27%). ^1^H NMR (400 MHz, DMSO-d_6_) δ 9.52 (s, 1H), 8.41 (s, 1H), 7.86 (s, 1H), 7.67 (s, 1H), 7.64 (s, 1H), 6.87 (s, 1H), 6.80 (d, *J* = 8.0 Hz, 1H), 6.62 (d, *J* = 8.0 Hz, 1H), 6.39 (s, 2H), 4.36 (s, 2H), 4.25 (s, 3H), 3.73 (s, 3H). ESI-MS: *m*/*z* = 324.1 [M + H]^+^.

Compounds **9**–**21** were synthesized from the intermediate (**1**-**2**) with various substituted arylboronic acids or arylboronates through Suzuki coupling reaction.

#### 3.2.19. 4-([1,3]Dioxolo[4,5-g]isoquinolin-8-yl)phenol (**9**)

Method A. White solid, yield 76%. ^1^H NMR (400 MHz, DMSO-d_6_) δ 9.69 (s, 1H), 8.99 (s, 1H), 8.18 (s, 1H), 7.54 (s, 1H), 7.30 (d, *J* = 8.5 Hz, 2H), 7.07 (s, 1H), 6.93 (d, *J* = 8.5 Hz, 2H), 6.20 (s, 2H). ESI-MS: *m*/*z* = 265.1 [M + H]^+^.

#### 3.2.20. 4-([1,3]Dioxolo[4,5-g]isoquinolin-8-yl)-2-(*tert*-butyl)phenol (**10**)

Method A. White solid, yield 70%. ^1^H NMR (400 MHz, DMSO-d_6_) δ 9.64 (s, 1H), 8.98 (s, 1H), 8.20 (s, 1H), 7.54 (s, 1H), 7.22 (d, *J* = 2.2 Hz, 1H), 7.15 (dd, *J* = 8.1, 2.2 Hz, 1H), 7.10 (s, 1H), 6.95 (d, *J* = 8.1 Hz, 1H), 6.20 (s, 2H), 1.39 (s, 9H). ESI-MS: *m*/*z* = 321.1 [M + H]^+^.

#### 3.2.21. 4-([1,3]Dioxolo[4,5-g]isoquinolin-8-yl)-2-(trifluoromethoxy)phenol (**11**)

Method A. White solid, yield 72%. ^1^H NMR (400 MHz, DMSO-d_6_) δ 9.02 (s, 1H), 8.22 (s, 2H), 7.57 (s, 1H), 7.38–7.33 (m, 2H), 7.19 (d, *J* = 8.3 Hz, 1H), 7.05 (s, 1H), 6.21 (s, 2H). ESI-MS: *m*/*z* = 349.1 [M + H]^+^.

#### 3.2.22. 4-([1,3]Dioxolo[4,5-g]isoquinolin-8-yl)-2-chlorophenol (**12**)

Method A. White solid, yield 60%. ^1^H NMR (400 MHz, DMSO-d_6_) δ 10.42 (s, 1H), 9.01 (s, 1H), 8.20 (s, 1H), 7.55 (s, 1H), 7.44 (d, *J* = 2.2 Hz, 1H), 7.27 (dd, *J* = 8.3, 2.2 Hz, 1H), 7.13 (d, *J* = 8.3 Hz, 1H), 7.05 (s, 1H), 6.21 (s, 2H). ESI-MS: *m*/*z* = 299.0 [M + H]^+^.

#### 3.2.23. 4-([1,3]Dioxolo[4,5-g]isoquinolin-8-yl)-2-(hydroxymethyl)phenol (**13**)

Method A. White solid, yield 65%. ^1^H NMR (400 MHz, DMSO-d_6_) δ 9.65 (s, 1H), 8.99 (s, 1H), 8.18 (s, 1H), 7.54 (s, 1H), 7.40 (d, *J* = 2.3 Hz, 1H), 7.18 (dd, *J* = 8.1, 2.3 Hz, 1H), 7.12 (s, 1H), 6.94 (d, *J* = 8.1 Hz, 1H), 6.20 (s, 2H), 5.04 (t, *J* = 5.7 Hz, 1H), 4.56 (d, *J* = 5.4 Hz, 2H). ESI-MS: *m*/*z* = 295.1 [M + H]^+^.

#### 3.2.24. 8-(3-Methoxyphenyl)-[1,3]dioxolo[4,5-g]isoquinoline (**14**)

Method A. White solid, yield 80%. ^1^H NMR (400 MHz, Chloroform-d) δ 8.99 (s, 1H), 8.33 (s, 1H), 7.46–7.36 (m, 1H), 7.19 (s, 1H), 7.02–6.97 (m, 2H), 6.09 (s, 2H), 3.87 (s, 3H). ESI-MS: *m*/*z* = 279.1 [M + H]^+^.

#### 3.2.25. 8-(4-Chloro-3-methoxyphenyl)-[1,3]dioxolo[4,5-g]isoquinoline (**15**)

Method A. White solid, yield 81%. ^1^H NMR (400 MHz, DMSO-d_6_) δ 9.06 (s, 1H), 8.28 (s, 1H), 7.62–7.54 (m, 2H), 7.24 (d, *J* = 1.9 Hz, 1H), 7.11 (s, 1H), 7.06 (dd, *J* = 7.9, 1.9 Hz, 1H), 6.22 (s, 2H), 3.90 (s, 3H). ESI-MS: *m*/*z* = 313.1 [M + H]^+^.

#### 3.2.26. 8-(3,4-Dimethoxyphenyl)-[1,3]dioxolo[4,5-g]isoquinoline (**16**)

Method A. White solid, yield 82%. ^1^H NMR (400 MHz, DMSO-d_6_) δ 9.01 (s, 1H), 8.24 (s, 1H), 7.56 (s, 1H), 7.14–7.10 (m, 2H), 7.05 (d, *J* = 2.0 Hz, 1H), 7.01 (dd, *J* = 8.1, 2.0 Hz, 1H), 6.21 (s, 2H), 3.83 (s, 3H), 3.80 (s, 3H). ESI-MS: *m*/*z* = 309.1 [M + H]^+^.

#### 3.2.27. 4-([1,3]Dioxolo[4,5-g]isoquinolin-8-yl)-2-methoxyaniline (**17**)

Method A. White solid, yield 60%. ^1^H NMR (400 MHz, DMSO-d_6_) δ 8.96 (s, 1H), 8.21 (s, 1H), 7.53 (s, 1H), 7.19 (s, 1H), 6.90 (d, *J* = 1.8 Hz, 1H), 6.83–6.77 (m, 2H), 6.21 (s, 2H), 4.95 (s, 2H), 3.81 (s, 3H). ESI-MS: *m*/*z* = 294.1 [M + H]^+^.

#### 3.2.28. 4-([1,3]Dioxolo[4,5-g]isoquinolin-8-yl)-2-methoxy-N-methylaniline (**18**)

Method A. White solid, yield 63%. ^1^H NMR (400 MHz, DMSO-d_6_) δ 8.97 (s, 1H), 8.23 (s, 1H), 7.53 (s, 1H), 7.21 (s, 1H), 7.00–6.89 (m, 2H), 6.62 (d, *J* = 8.0 Hz, 1H), 6.20 (s, 2H), 5.28–5.20 (m, 1H), 3.83 (s, 3H), 2.79 (d, *J* = 4.3 Hz, 3H). ESI-MS: *m*/*z* = 308.1 [M + H]^+^.

#### 3.2.29. 3-([1,3]Dioxolo[4,5-g]isoquinolin-8-yl)phenol (**19**)

Method A. White solid, yield 70%. M.p. 262–264 °C. ^1^H NMR (400 MHz, DMSO-d_6_) δ 9.67 (s, 1H), 9.03 (s, 1H), 8.21 (s, 1H), 7.56 (s, 1H), 7.34 (t, *J* = 7.8 Hz, 1H), 7.07 (s, 1H), 6.91–6.81 (m, 3H), 6.21 (s, 2H). ^13^C NMR (126 MHz, DMSO) δ 157.6, 151.2, 149.5, 148.1, 141.5, 138.1, 132.2, 131.5, 129.9, 125.6, 120.3, 116.5, 114.9, 103.3, 102.1, 99.8. HRMS (ESI+) calcd for C_16_H_12_NO_3_+ (M + H^+^): 266.0812, found: 266.0812.

#### 3.2.30. 4-([1,3]Dioxolo[4,5-g]isoquinolin-8-yl)-2-chlorophenol (**20**)

Method A. White solid, yield 65%. M.p. 229–231 °C. ^1^H NMR (500 MHz, DMSO-d_6_) δ 9.04 (s, 1H), 8.21 (s, 1H), 7.57 (s, 1H), 7.49 (d, *J* = 8.1 Hz, 1H), 7.09 (s, 1H), 7.06 (d, *J* = 2.0 Hz, 1H), 6.92 (dd, *J* = 8.1, 2.0 Hz, 1H), 6.22 (s, 2H). ^13^C NMR (126 MHz, DMSO) δ 153.2, 151.4, 149.8, 148.1, 141.5, 136.7, 131.4, 131.2, 130.2, 125.6, 121.3, 119.5, 117.7, 103.4, 102.2, 99.8. HRMS (ESI+) calcd for C_16_H_11_ClNO_3_+ (M + H^+^): 300.0422, found: 300.0424. HPLC analysis: 12.868 min, 96.5% purity.

#### 3.2.31. 8-(Benzo[d][1,3]dioxol-5-yl)-[1,3]dioxolo[4,5-g]isoquinoline (**21**)

Method A. White solid, yield 78%. ^1^H NMR (400 MHz, DMSO-d_6_) δ 9.01 (s, 1H), 8.21 (s, 1H), 7.56 (s, 1H), 7.12–7.04 (m, 3H), 6.94 (dd, *J* = 7.9, 1.8 Hz, 1H), 6.21 (s, 2H), 6.12 (s, 2H). ESI-MS: *m*/*z* = 293.1 [M + H]^+^.

#### 3.2.32. 2-Chloro-5-(isoquinolin-4-yl)phenol (**22**)

Method A. Compound 22 was synthesized from commercially available reagent (4-1); white solid, yield 70%. ^1^H NMR (400 MHz, DMSO-d_6_) δ 10.48 (s, 1H), 9.35 (s, 1H), 8.42 (s, 1H), 8.23 (d, *J* = 8.0 Hz, 1H), 7.88 (d, *J* = 8.4 Hz, 1H), 7.85–7.80 (m, 1H), 7.78–7.71 (m, 1H), 7.52 (dd, *J* = 8.0, 1.1 Hz, 1H), 7.11 (s, 1H), 7.00–6.92 (m, 1H). ESI-MS: *m*/*z* = 255.1 [M + H]^+^.

#### 3.2.33. Synthesis of Intermediate (**4**-**3**)

To a solution of commercially available reagent (**4**-**2**) (500 mg, 3.4 mmol) in acetic acid (30 mL) was added NBS (787 mg, 4.4 mmol), and the reaction was stirred at 90 °C for 12 h. Upon completion of the reaction, the mixture was cooled to room temperature. The solvent was removed in vacuo, and NaHCO_3_ (aq) was added to adjust the pH to 8–9. The resulting mixture was diluted with EtOAc (30 mL) and water (15 mL). The organic layer was separated and washed with brine (15 mL × 2), dried over Na_2_SO_4_, filtrated, and evaporated under reduced pressure to afford (**4**-**3**) as a yellow solid (306 mg, 40%). ^1^H NMR (400 MHz, DMSO-d_6_) δ 9.34 (s, 1H), 8.78 (s, 1H), 8.45–8.30 (m, 1H), 7.80–7.63 (m, 2H). ESI-MS: *m*/*z* = 226.0 [M + H]^+^.

#### 3.2.34. Synthesis of Intermediate (**4**-**6**)

To a solution of commercially available reagent (**4**-**5**) (500 mg, 3.4 mmol) in acetic acid (30 mL) was added NBS (787 mg, 4.4 mmol), and the reaction was stirred at 90 °C for 12 h. Upon completion of the reaction, the mixture was cooled to room temperature. The solvent was removed in vacuo, and NaHCO_3_ (aq) was added to adjust the pH to 8–9. The resulting mixture was diluted with EtOAc (30 mL) and water (15 mL). The organic layer was separated and washed with brine (15 mL × 2), dried over Na_2_SO_4_, filtrated, and evaporated under reduced pressure to afford (**4**-**6**) as a yellow solid (344 mg, 45%). ^1^H NMR (400 MHz, DMSO-d_6_) δ 9.31 (s, 1H), 8.75 (s, 1H), 8.18 (dd, *J* = 9.3, 5.2 Hz, 1H), 8.07 (dd, *J* = 9.2, 2.7 Hz, 1H), 7.90 (td, *J* = 9.1, 2.6 Hz, 1H). ESI-MS: *m*/*z* = 226.0 [M + H]^+^.

#### 3.2.35. 2-Chloro-5-(6-ethoxyisoquinolin-4-yl)phenol (**23**)

To a solution of (**4**-**3**) (100 mg, 0.44 mmol) in dry EtOH (10 mL) was added NaOEt (60 mg, 0.88 mmol). The reaction was stirred at room temperature for 12 h. Upon completion of the reaction, the volatiles were removed in vacuo. The resulting mixture was then diluted with EtOAc (20 mL) and water (10 mL). The organic layer was separated and washed with brine (10 mL × 2), dried over Na_2_SO_4_, filtrated, and evaporated under reduced pressure. The residue was purified by flash chromatography on silica gel (10–50% EA in PE) to give 4-bromo-6-ethoxyisoquinoline (**4**-**4**-**23**) as a yellow solid (67 mg, 60%). ^1^H NMR (400 MHz, DMSO-d_6_) δ 9.16 (s, 1H), 8.66 (s, 1H), 8.13 (d, *J* = 8.9 Hz, 1H), 7.42 (dd, *J* = 8.9, 2.4 Hz, 1H), 7.31 (d, *J* = 2.4 Hz, 1H), 4.27 (q, *J* = 7.0 Hz, 2H), 1.43 (t, *J* = 7.0 Hz, 3H). ESI-MS: *m*/*z* = 252.0 [M + H]^+^. Method A. The intermediate (**4**-**4**-**23**) was reacted with (4-chloro-3-hydroxyphenyl)boronic acid through Suzuki coupling reaction to afford **23** as a white solid (65%). M.p. 196–198 °C. ^1^H NMR (400 MHz, DMSO-d_6_) δ 9.17 (s, 1H), 8.30 (s, 1H), 8.22 (s, 1H), 8.13 (d, *J* = 9.0 Hz, 1H), 7.50 (d, *J* = 8.1 Hz, 1H), 7.36 (d, *J* = 9.0 Hz, 1H), 7.16–7.10 (m, 2H), 6.99 (d, *J* = 8.1 Hz, 1H), 4.07 (q, *J* = 7.0 Hz, 2H), 1.35 (t, *J* = 7.0 Hz, 3H). ^13^C NMR (126 MHz, DMSO) δ 160.3, 153.3, 151.3, 142.8, 136.6, 134.9, 130.3, 123.8, 121.3, 120.0, 119.5, 117.7, 102.8, 63.5, 14.4. HRMS (ESI+) calcd for C_17_H_15_ClNO_2_+ (M + H^+^): 300.0786, found: 300.0788. HPLC analysis: 14.827 min, 99.1% purity.

#### 3.2.36. 2-Chloro-5-(7-ethoxyisoquinolin-4-yl)phenol (**24**)

To a solution of (**4**-**6**) (100 mg, 0.44 mmol) in dry EtOH (10 mL) was added NaOEt (60 mg, 0.88 mmol). The reaction was stirred at room temperature for 12 h. Upon completion of the reaction, the volatiles were removed in vacuo. The resulting mixture was then diluted with EtOAc (20 mL) and water (10 mL). The organic layer was separated and washed with brine (10 mL × 2), dried over Na_2_SO_4_, filtrated, and evaporated under reduced pressure. The residue was purified by flash chromatography on silica gel (10–50% EA in PE) to give 4-bromo-6-ethoxyisoquinoline (**4**-**7**-**24**) as a white solid (61 mg, 55%). ^1^H NMR (400 MHz, Chloroform-d) δ 9.23 (s, 1H), 9.14 (s, 1H), 7.73 (d, *J* = 9.0 Hz, 1H), 7.35 (dd, *J* = 9.0, 2.5 Hz, 1H), 7.21 (d, *J* = 2.5 Hz, 1H), 4.18 (q, *J* = 7.0 Hz, 2H), 1.50 (t, *J* = 7.0 Hz, 3H). ESI-MS: *m*/*z* = 252.0 [M + H]^+^. Method A. The intermediate (**4**-**7**-**24**) was reacted with (4-chloro-3-hydroxyphenyl)boronic acid through Suzuki coupling reaction to afford **24** as a white solid (60%). ^1^H NMR (400 MHz, DMSO-d_6_) δ 10.56 (s, 1H), 9.30 (s, 1H), 7.87 (d, *J* = 9.2 Hz, 1H), 7.69 (s, 1H), 7.59 (d, *J* = 8.2 Hz, 1H), 7.55–7.48 (m, 1H), 7.17 (s, 1H), 7.07–6.99 (m, 1H), 4.30 (q, *J* = 7.0 Hz, 2H), 1.51 (t, *J* = 7.0 Hz, 3H). ESI-MS: *m*/*z* = 300.07 [M + H]^+^.

#### 3.2.37. 4-(4-Chloro-3-hydroxyphenyl)isoquinoline-6-carboxylic Acid (**25**)

To a solution of commercially available reagent methyl isoquinoline-6-carboxylate (200 mg, 1.1 mmol) in acetic acid (30 mL) was added NBS (393 mg, 2.2 mmol); the reaction was stirred at 90 °C for 12 h. Upon completion of the reaction, the mixture was cooled to room temperature. The solvent was removed in vacuo, and NaHCO_3_ (aq) was added to adjust the pH to 8–9. The resulting mixture was diluted with EtOAc (30 mL) and water (15 mL). The organic layer was separated and washed with brine (15 mL × 2), dried over Na_2_SO_4_, filtrated, and evaporated under reduced pressure to afford methyl 4-bromoisoquinoline-6-carboxylate (**4**-**12**-**25**) as a yellow solid (88 mg, 30%). ^1^H NMR (400 MHz, DMSO-d_6_) δ 9.46 (s, 1H), 8.89 (s, 1H), 8.69 (s, 1H), 8.38 (d, *J* = 8.6 Hz, 1H), 8.26 (d, *J* = 8.6 Hz, 1H), 3.98 (s, 3H). ESI-MS: *m*/*z* = 266.0 [M + H]^+^. Method A. The intermediate (**4**-**12**-**25**) was reacted with (4-chloro-3-hydroxyphenyl)boronic acid through Suzuki coupling reaction to afford **25** as a white solid (30%). ^1^H NMR (400 MHz, DMSO-d_6_) δ 10.57 (s, 1H), 9.45 (s, 1H), 8.51 (s, 1H), 8.47 (s, 1H), 8.32 (d, *J* = 8.4 Hz, 1H), 8.18 (d, *J* = 8.5 Hz, 1H), 7.55 (d, *J* = 8.1 Hz, 1H), 7.12 (d, *J* = 2.1 Hz, 1H), 6.99 (dd, *J* = 8.1, 2.1 Hz, 1H). ESI-MS: *m*/*z* = 300.0 [M + H]^+^.

#### 3.2.38. 4-(4-Chloro-3-hydroxyphenyl)isoquinoline-7-carboxylic Acid (**26**)

To a solution of commercially available reagent methyl 4-bromoisoquinoline-7-carboxylate (200 mg, 1.1 mmol) in acetic acid (30 mL) was added NBS (393 mg, 2.2 mmol); the reaction was stirred at 90 °C for 12 h. Upon completion of the reaction, the mixture was cooled to room temperature. The solvent was removed in vacuo, and NaHCO_3_ (aq) was added to adjust the pH to 8–9. The resulting mixture was diluted with EtOAc (30 mL) and water (15 mL). The organic layer was separated and washed with brine (15 mL × 2), dried over Na_2_SO_4_, filtrated, and evaporated under reduced pressure to afford methyl 4-bromoisoquinoline-6-carboxylate (**4**-**12**-**26**) as a yellow solid (103 mg, 35%). ^1^H NMR (400 MHz, DMSO-d_6_) δ 9.55 (s, 1H), 8.92 (s, 1H), 8.90 (s, 1H), 8.40 (d, *J* = 8.9 Hz, 1H), 8.22 (d, *J* = 8.9 Hz, 1H), 3.96 (s, 3H). ESI-MS: *m*/*z* = 266.0 [M + H]^+^. Method A. The intermediate (**4**-**12**-**26**) was reacted with (4-chloro-3-hydroxyphenyl)boronic acid through Suzuki coupling reaction to afford **26** as a white solid (33%). ^1^H NMR (400 MHz, DMSO-d_6_) δ 10.52 (s, 1H), 9.53 (s, 1H), 8.87 (d, *J* = 1.7 Hz, 1H), 8.52 (s, 1H), 8.24 (dd, *J* = 8.9, 1.8 Hz, 1H), 7.95 (d, *J* = 8.9 Hz, 1H), 7.53 (d, *J* = 8.1 Hz, 1H), 7.11 (d, *J* = 2.0 Hz, 1H), 6.99 (dd, *J* = 8.1, 2.0 Hz, 1H). ESI-MS: *m*/*z* = 300.0 [M + H]^+^.

#### 3.2.39. 2-Chloro-5-(7-(2-methoxyethoxy)isoquinolin-4-yl)phenol (**27**)

Method C. To a solution of NaH (41 mg, 1.7 mmol) in dry DMF at 0 °C was added 2-methoxyethan-1-ol (100 mg, 1.3 mmol). After stirring for 0.5h at the same temperature, the intermediate (**4**-**6**) (326 mg, 1.4 mmol) was added followed by recovery to room temperature and stirring for 12 h. Upon completion of the reaction, cold water (5 mL) was added to quench the mixture, and EtOAc (30 mL) and water (15 mL) were added. The organic layer was separated and washed with brine (15 mL × 2), dried over Na_2_SO_4_, filtrated, and evaporated under reduced pressure to afford 4-bromo-7-(2-methoxyethoxy)isoquinoline (**4**-**7**-**27**) as a yellow solid (257 mg, 65%). ^1^H NMR (400 MHz, DMSO-d_6_) δ 9.19 (s, 1H), 8.59 (s, 1H), 8.07–7.99 (m, 2H), 7.67–7.59 (m, 1H), 4.29 (t, *J* = 4.6 Hz, 2H), 3.75 (t, *J* = 4.6 Hz, 2H), 3.35 (s, 3H). ESI-MS: *m*/*z* = 282.0 [M + H]^+^. Method A. The intermediate (**4**-**7**-**27**) was reacted with (4-chloro-3-hydroxyphenyl)boronic acid through Suzuki coupling reaction to afford **27** as a white solid (70%). ^1^H NMR (400 MHz, DMSO-d_6_) δ 9.20 (s, 1H), 8.36 (s, 1H), 8.26 (s, 1H), 7.78 (d, *J* = 9.2 Hz, 1H), 7.62 (d, *J* = 2.7 Hz, 1H), 7.50 (d, *J* = 8.1 Hz, 1H), 7.46 (dd, *J* = 9.2, 2.7 Hz, 1H), 7.08 (d, *J* = 2.1 Hz, 1H), 6.94 (dd, *J* = 8.1, 2.1 Hz, 1H), 4.28 (t, *J* = 4.0 Hz, 2H), 3.75 (t, *J* = 4.0 Hz, 2H), 3.34 (s, 3H). ESI-MS: *m*/*z* = 330.1 [M + H]^+^

#### 3.2.40. Synthesis of Intermediate (**4**-**9**)

To a solution of commercially available (**4**-**8**) (100 mg, 0.61 mmol) in dry EtOH (10 mL) was added NaOEt (92 mg, 1.34 mmol). The reaction was stirred at room temperature for 12 h. Upon completion of the reaction, the volatiles were removed in vacuo. The resulting mixture was then diluted with EtOAc (20 mL) and water (10 mL). The organic layer was separated and washed with brine (10 mL × 2), dried over Na_2_SO_4_, filtrated, and evaporated under reduced pressure. The residue was purified by flash chromatography on silica gel (0–20% EA in PE) to give (**4**-**9**) as a white solid (100 mg, 75%). ^1^H NMR (400 MHz, Chloroform-d) δ 9.01 (s, 1H), 8.35 (d, *J* = 5.7 Hz, 1H), 7.48 (d, *J* = 5.7 Hz, 1H), 7.19 (s, 1H), 7.05 (s, 1H), 4.28–4.16 (m, 4H), 1.56 (td, *J* = 7.0, 1.4 Hz, 6H). ESI-MS: *m*/*z* = 218.1 [M + H]^+^.

#### 3.2.41. Synthesis of Intermediate (**4**-**10**)

To a solution of intermediate (**4**-**9**) (100 mg, 0.45 mmol) in acetic acid (30 mL) was added NBS (122 mg, 0.68 mmol); the reaction was stirred at 90 °C for 12 h. Upon completion of the reaction, the mixture was cooled to room temperature. The solvent was removed in vacuo, and NaHCO_3_ (aq) was added to adjust the pH to 8–9. The resulting mixture was diluted with EtOAc (30 mL) and water (15 mL). The organic layer was separated and washed with brine (15 mL × 2), dried over Na_2_SO_4_, filtrated, and evaporated under reduced pressure to afford (**4**-**10**) as a yellow solid (60 mg, 45%). ^1^H NMR (400 MHz, Chloroform-d) δ 9.05 (s, 1H), 8.48 (d, *J* = 5.9 Hz, 1H), 7.90 (d, *J* = 5.9 Hz, 1H), 7.23 (s, 1H), 4.29–4.21 (m, 4H), 1.53–1.45 (m, 6H). ESI-MS: *m*/*z* = 296.0 [M + H]^+^.

#### 3.2.42. 2-Chloro-5-(7-(2-methoxyethoxy)isoquinolin-4-yl)phenol (**28**)

Method A. The intermediate (**4**-**10**) was reacted with (4-chloro-3-hydroxyphenyl)boronic acid through Suzuki coupling reaction to afford **28** as a white solid (72%). M.p. 195–197 °C. ^1^H NMR (400 MHz, DMSO-d_6_) δ 10.32 (s, 1H), 9.16 (s, 1H), 8.25 (d, *J* = 5.6 Hz, 1H), 7.64 (s, 1H), 7.47 (d, *J* = 8.4 Hz, 1H), 7.16 (d, *J* = 5.6 Hz, 1H), 6.90 (s, 1H), 6.76 (d, *J* = 8.4 Hz, 1H), 4.26 (q, *J* = 7.0 Hz, 2H), 3.95 (q, *J* = 7.0 Hz, 2H), 1.46 (t, *J* = 7.0 Hz, 3H), 1.06 (t, *J* = 7.0 Hz, 3H). HRMS (ESI+) calcd for C_18_H_16_NO4+ (M + H^+^): 310.1074, found: 310.1072.

#### 3.2.43. 2-Chloro-4-(6-propoxyisoquinolin-4-yl)phenol (**29**)

Method C. Intermediate 4-bromo-6-propoxyisoquinoline (**4**-**4**-**29**) was obtained as a white solid (yield 60%). ^1^H NMR (400 MHz, DMSO-d_6_) δ 9.16 (s, 1H), 8.66 (s, 1H), 8.13 (d, *J* = 9.0 Hz, 1H), 7.43 (dd, *J* = 9.0, 2.4 Hz, 1H), 7.31 (d, *J* = 2.4 Hz, 1H), 4.17 (t, *J* = 6.5 Hz, 2H), 1.90–1.76 (m, 2H), 1.04 (t, *J* = 7.4 Hz, 3H). ESI-MS: *m*/*z* = 266.0 [M + H]^+^. Method A. The intermediate (**4**-**4**-**29**) was reacted with (4-chloro-3-hydroxyphenyl)boronic acid through Suzuki coupling reaction to afford **29** as a white solid (70%). ^1^H NMR (400 MHz, DMSO-d_6_) δ 9.17 (s, 1H), 8.33 (s, 1H), 8.30 (s, 1H), 8.13 (d, *J* = 9.0 Hz, 1H), 7.50 (d, *J* = 8.1 Hz, 1H), 7.37 (dd, *J* = 9.0, 2.4 Hz, 1H), 7.16–7.11 (m, 2H), 6.98 (dd, *J* = 8.1, 2.1 Hz, 1H), 3.98 (t, *J* = 6.4 Hz, 2H), 1.79–1.72 (m, 2H), 0.98 (t, *J* = 7.4 Hz, 3H). ESI-MS: *m*/*z* = 314.1 [M + H]^+^.

#### 3.2.44. 2-Chloro-4-(6-cyclopropoxyisoquinolin-4-yl)phenol (**30**)

Method C. Intermediate 4-bromo-6-cyclopropoxyisoquinoline (**4**-**4**-**30**) was obtained as a white solid (yield 50%). ^1^H NMR (400 MHz, Chloroform-d) δ 9.01 (s, 1H), 8.64 (s, 1H), 7.85 (d, *J* = 9.0 Hz, 1H), 7.70 (d, *J* = 2.4 Hz, 1H), 7.30–7.22 (m, 1H), 3.97–3.90 (m, 1H), 0.97–0.91 (m, 2H), 0.89–0.80 (m, 2H). ESI-MS: *m*/*z* = 264.0 [M + H]^+^. Method A. The intermediate (**4**-**4**-**30**) was reacted with (4-chloro-3-hydroxyphenyl)boronic acid through Suzuki coupling reaction to afford **30** as a white solid (65%). ^1^H NMR (400 MHz, DMSO-d_6_) δ 10.48 (s, 1H), 9.19 (s, 1H), 8.33 (s, 1H), 8.15 (d, *J* = 8.9 Hz, 1H), 7.51 (d, *J* = 8.1 Hz, 1H), 7.48 (d, *J* = 2.4 Hz, 1H), 7.39 (dd, *J* = 8.9, 2.4 Hz, 1H), 7.14 (d, *J* = 2.1 Hz, 1H), 7.01 (dd, *J* = 8.1, 2.1 Hz, 1H), 3.95–3.86 (m, 1H), 0.81–0.76 (m, 2H), 0.73–0.68 (m, 2H). ESI-MS: *m*/*z* = 312.1 [M + H]^+^.

#### 3.2.45. 2-Chloro-4-(6-isopropoxyisoquinolin-4-yl)phenol (**31**)

Method C. Intermediate 4-bromo-6-isopropoxyisoquinoline (**4**-**4**-**31**) was obtained as a white solid (yield 55%). ^1^H NMR (400 MHz, Chloroform-d) δ 9.01 (s, 1H), 8.64 (s, 1H), 7.85 (d, *J* = 9.0 Hz, 1H), 7.70 (d, *J* = 2.4 Hz, 1H), 7.30–7.22 (m, 1H), 4.78–4.68 (m, 1H), 1.42 (d, *J* = 6.0 Hz, 2H). ESI-MS: *m*/*z* = 266.0 [M + H]^+^. Method A. The intermediate (**4**-**4**-**31**) was reacted with (4-chloro-3-hydroxyphenyl)boronic acid through Suzuki coupling reaction to afford **31** as a white solid (70%). ^1^H NMR (400 MHz, DMSO-d_6_) δ 10.46 (s, 1H), 9.16 (s, 1H), 8.29 (s, 1H), 8.13 (d, *J* = 9.0 Hz, 1H), 7.56–7.48 (m, 1H), 7.38–7.30 (m, 1H), 7.10 (s, 2H), 6.97 (d, *J* = 7.2 Hz, 1H), 4.69–4.60 (m, 1H), 1.29 (d, *J* = 5.9 Hz, 6H). ESI-MS: *m*/*z* = 314.1 [M + H]^+^.

#### 3.2.46. 2-Chloro-4-(6-(2-hydroxyethoxy)isoquinolin-4-yl)phenol (**32**)

Method C. Intermediate 2-((4-bromoisoquinolin-6-yl)oxy)ethan-1-ol (**4**-**4**-**32**) was obtained as a white solid (yield 35%). ^1^H NMR (400 MHz, DMSO-d_6_) δ 9.18 (s, 1H), 8.68 (s, 1H), 8.15 (d, *J* = 9.0 Hz, 1H), 7.44 (dd, *J* = 9.0, 2.4 Hz, 1H), 7.33 (d, *J* = 2.4 Hz, 1H), 5.02 (s, 1H), 4.23 (t, *J* = 4.8 Hz, 2H), 3.82 (t, *J* = 4.8 Hz, 2H). ESI-MS: *m*/*z* = 268.0 [M + H]^+^. Method A. The intermediate (**4**-**4**-**32**) was reacted with (4-chloro-3-hydroxyphenyl)boronic acid through Suzuki coupling reaction to afford **32** as a white solid (50%). ^1^H NMR (400 MHz, DMSO-d_6_) δ 9.18 (s, 1H), 8.31 (s, 1H), 8.19 (s, 1H), 8.14 (d, *J* = 9.0 Hz, 1H), 7.50 (d, *J* = 8.1 Hz, 1H), 7.39 (dd, *J* = 9.0, 2.4 Hz, 1H), 7.16 (d, *J* = 2.4 Hz, 1H), 7.13 (d, *J* = 2.1 Hz, 1H), 6.99 (dd, *J* = 8.1, 2.1 Hz, 1H), 4.92 (s, 1H), 4.04 (t, *J* = 4.9 Hz, 2H), 3.74 (t, *J* = 4.9 Hz, 2H). ESI-MS: *m*/*z* = 316.1 [M + H]^+^.

#### 3.2.47. 2-Chloro-4-(6-(2-methoxyethoxy)isoquinolin-4-yl)phenol (**33**)

Method C. Intermediate 4-bromo-6-(2-methoxyethoxy)isoquinoline (**4**-**4**-**33**) was obtained as a white solid (yield 50%). ^1^H NMR (400 MHz, Chloroform-d) δ 9.02 (s, 1H), 8.65 (s, 1H), 7.87 (d, *J* = 8.9 Hz, 1H), 7.39 (d, *J* = 2.6 Hz, 2H), 7.34 (dd, *J* = 8.9, 2.6 Hz, 1H), 4.41–4.27 (m, 2H), 3.96–3.76 (m, 2H), 3.50 (s, 3H). ESI-MS: *m*/*z* = 282.0 [M + H]^+^. Method A. The intermediate (**4**-**4**-**33**) was reacted with (4-chloro-3-hydroxyphenyl)boronic acid through Suzuki coupling reaction to afford **33** as a white solid (55%). ^1^H NMR (400 MHz, DMSO-d_6_) δ 10.42 (s, 1H), 9.18 (s, 1H), 8.31 (s, 1H), 8.14 (d, *J* = 9.0 Hz, 1H), 7.50 (d, *J* = 8.1 Hz, 1H), 7.39 (dd, *J* = 9.0, 2.4 Hz, 1H), 7.16 (d, *J* = 2.4 Hz, 1H), 7.13 (d, *J* = 2.1 Hz, 1H), 6.99 (dd, *J* = 8.1, 2.1 Hz, 1H), 4.22–4.07 (m, 2H), 3.72–3.57 (m, 2H), 3.30 (s, 3H). ESI-MS: *m*/*z* = 330.1 [M + H]^+^.

#### 3.2.48. 2-Chloro-5-(6-(ethylamino)isoquinolin-4-yl)phenol (**34**)

Method C. Intermediate 4-bromo-6-(2-methoxyethoxy)isoquinoline (**4**-**4**-**34**) was obtained as a white solid (yield 52%). M.p. 215-217 °C. ^1^H NMR (400 MHz, DMSO-d_6_) δ 8.83 (s, 1H), 8.42 (s, 1H), 7.81 (d, *J* = 8.9 Hz, 1H), 7.13 (dd, *J* = 8.9, 2.2 Hz, 1H), 6.92 (t, *J* = 5.0 Hz, 1H), 6.66 (d, *J* = 2.2 Hz, 1H), 3.23–3.09 (m, 2H), 1.25 (t, *J* = 7.2 Hz, 3H). ESI-MS: *m*/*z* = 251.0 [M + H]^+^. Method A. The intermediate (**4**-**4**-**34**) was reacted with (4-chloro-3-hydroxyphenyl)boronic acid through Suzuki coupling reaction to afford **34** as a white solid (54%). ^1^H NMR (400 MHz, DMSO-d_6_) δ 10.36 (s, 1H), 8.86 (s, 1H), 8.06 (s, 1H), 7.83 (d, *J* = 8.9 Hz, 1H), 7.46 (d, *J* = 8.1 Hz, 1H), 7.13–6.98 (m, 2H), 6.93 (dd, *J* = 8.1, 2.0 Hz, 1H), 6.63–6.47 (m, 2H), 3.07–2.92 (m, 2H), 1.16 (t, *J* = 7.1 Hz, 3H). ^13^C NMR (126 MHz, DMSO) δ 153.6, 151.1, 151.0, 143.0, 138.0, 136.2, 130.5, 129.7, 129.5, 122.2, 121.7, 119.4, 119.0, 118.1, 97.9, 37.4, 14.3. HRMS (ESI+) calcd for C_17_H_16_ClN_2_O+ (M + H^+^): 299.0947, found: 299.0946.

#### 3.2.49. *Tert*-Butyl 3-((4-(4-chloro-3-hydroxyphenyl)isoquinolin-6-yl)oxy)pyrrolidine-1-carboxylate (**35**)

Method C. Intermediate *tert*-butyl 3-((4-bromoisoquinolin-6-yl)oxy)pyrrolidine-1-carboxylate (**4**-**4**-**35**) was obtained as a white solid (yield 50%). ^1^H NMR (400 MHz, Chloroform-d) δ 9.02 (s, 1H), 8.65 (s, 1H), 7.88 (d, *J* = 8.9 Hz, 1H), 7.33 (d, *J* = 2.3 Hz, 1H), 7.27–7.18 (m, 1H), 5.14 (s, 1H), 3.67–3.54 (m, 2H), 2.39–2.19 (m, 2H), 1.80–1.62 (m, 2H), 1.47 (s, 9H). ESI-MS: *m*/*z* = 393.1 [M + H]^+^. Method A. The intermediate (**4**-**4**-**35**) was reacted with (4-chloro-3-hydroxyphenyl)boronic acid through Suzuki coupling reaction to afford **35** as a white solid (50%). ^1^H NMR (400 MHz, DMSO-d_6_) δ 10.45 (s, 1H), 9.20 (s, 1H), 8.32 (s, 1H), 8.17 (d, *J* = 9.0 Hz, 1H), 7.50 (d, *J* = 8.2 Hz, 1H), 7.41 (dd, *J* = 9.1, 2.1 Hz, 1H), 7.13–7.07 (m, 2H), 6.97 (dd, *J* = 8.2, 2.1 Hz, 1H), 5.16–4.92 (m, 1H), 3.59–3.51 (m, 1H), 3.45–3.37 (m, 3H), 2.19–1.98 (m, 2H), 1.39 (s, 9H). ESI-MS: *m*/*z* = 441.2 [M + H]^+^.

#### 3.2.50. 2-Chloro-5-(6-(pyrrolidin-3-yloxy)isoquinolin-4-yl)phenol (**36**)

To a solution of **35** (100 mg, 0.23 mmol) in EtOAc (10 mL) at 0 °C was added 5 mL of HCl (aq, 4M in EtOAc). The reaction was stirred at room temperature for 2 h. Upon completion of the reaction, the volatiles were removed in vacuo, and NaHCO_3_ (aq) was added to adjust the pH to 8~9. The resulting mixture was diluted with EtOAc (30 mL) and water (15 mL). The organic layer was separated and washed with brine (15 mL × 2), dried over Na_2_SO_4_, filtrated, and evaporated under reduced pressure. The residue was purified by flash chromatography on silica gel (0–5% MeOH in DCM) to give **36** as a white solid (71 mg, 90%). ^1^H NMR (400 MHz, DMSO-d_6_) δ 9.68 (s, 1H), 9.60–9.48 (m, 2H), 8.53 (s, 1H), 7.69 (dd, *J* = 9.2, 2.3 Hz, 1H), 7.57 (d, *J* = 8.1 Hz, 1H), 7.30–7.25 (m, 2H), 7.04 (dd, *J* = 8.1, 2.0 Hz, 1H), 5.45–5.38 (m, 1H), 3.83–3.74 (m, 2H), 3.18–3.12 (m, 2H), 2.33–2.12 (m, 2H). ESI-MS: *m*/*z* = 341.2 [M + H]^+^.

#### 3.2.51. 6-Ethoxy-4-phenylisoquinoline (**37**)

Intermediate (**5**-**1**) was prepared as previously described for the synthesis of compound **23**. Method A. The intermediate (**5**-**1**) was reacted with phenylboronic acid through Suzuki coupling reaction to afford **37** as a white solid (80%). M.p. 56–58 °C. ^1^H NMR (400 MHz, DMSO-d_6_) δ 9.18 (s, 1H), 8.33 (s, 1H), 8.14 (d, *J* = 8.9 Hz, 1H), 7.62–7.51 (m, 5H), 7.36 (dd, *J* = 9.0, 2.3 Hz, 1H), 7.10 (d, *J* = 2.3 Hz, 1H), 4.03 (q, *J* = 6.9 Hz, 2H), 1.33 (t, *J* = 6.9 Hz, 3H). ^13^C NMR (126 MHz, DMSO) δ 160.2, 151.1, 143.0, 136.7, 135.1, 131.6, 130.1, 129.7, 128.8, 128.0, 123.8, 119.9, 102.9, 63.4, 14.4. HRMS (ESI+) calcd for C_17_H_16_NO+ (M + H^+^): 250.1226, found: 250.1229.

#### 3.2.52. 4-(4-Chlorophenyl)-6-ethoxyisoquinoline (**38**)

Method A. The intermediate (**5**-**1**) was reacted with (4-chlorophenyl)boronic acid through Suzuki coupling reaction to afford **38** as a white solid (82%). M.p. 80–82 °C. ^1^H NMR (400 MHz, DMSO-d_6_) δ 9.20 (s, 1H), 8.33 (d, *J* = 2.0 Hz, 1H), 8.15 (dd, *J* = 9.0, 2.0 Hz, 1H), 7.68–7.56 (m, 4H), 7.37 (d, *J* = 9.0 Hz, 1H), 7.07 (s, 1H), 4.06 (q, *J* = 6.9 Hz, 2H), 1.35 (t, *J* = 6.9 Hz, 3H). ^13^C NMR (126 MHz, DMSO) δ 160.3, 151.4, 143.1, 135.6, 134.9, 132.8, 131.5, 130.4, 130.2, 128.9, 123.8, 120.0, 102.6, 63.5, 14.4. HRMS (ESI+) calcd for C_17_H_15_ClNO+ (M + H^+^): 284.0837, found: 284.0838.

#### 3.2.53. 3-(6-Ethoxyisoquinolin-4-yl)phenol (**39**)

Method A. The intermediate (**5**-**1**) was reacted with (3-hydroxyphenyl)boronic acid through Suzuki coupling reaction to afford **39** as a white solid (65%). M.p. 210–212 °C. ^1^H NMR (400 MHz, DMSO-d_6_) δ 9.66 (s, 1H), 9.16 (s, 1H), 8.30 (s, 1H), 8.13 (d, *J* = 9.0 Hz, 1H), 7.41–7.28 (m, 2H), 7.17 (s, 1H), 6.98–6.85 (m, 3H), 4.06 (q, *J* = 7.2 Hz, 2H), 1.39–1.27 (t, *J* = 7.2 Hz, 3H). ^13^C NMR (126 MHz, DMSO) δ 160.1, 157.6, 151.0, 142.7, 138.0, 135.1, 131.6, 130.1, 129.9, 123.8, 120.3, 119.8, 116.4, 114.9, 103.1, 63.4, 14.4. HRMS (ESI+) calcd for C_17_H_16_NO_2_+ (M + H^+^): 266.1176, found: 266.1177.

#### 3.2.54. 5-(6-Ethoxyisoquinolin-4-yl)-2-methylphenol (**40**)

Method A. The intermediate (**5**-**1**) was reacted with (3-hydroxy-4-methylphenyl)boronic acid through Suzuki coupling reaction to afford **40** as a white solid (63%). M.p. 199–201 °C. ^1^H NMR (400 MHz, DMSO-d_6_) δ 9.53 (s, 1H), 9.14 (s, 1H), 8.27 (s, 1H), 8.11 (d, *J* = 9.0 Hz, 1H), 7.34 (dd, *J* = 9.0, 2.3 Hz, 1H), 7.23 (d, *J* = 7.7 Hz, 1H), 7.20 (s, 1H), 6.95 (s, 1H), 6.88 (d, *J* = 7.7 Hz, 1H), 4.05 (q, *J* = 6.9 Hz, 2H), 1.35 (t, *J* = 6.9 Hz, 3H). ^13^C NMR (126 MHz, DMSO) δ 160.5, 156.0, 151.2, 143.2, 135.6, 135.5, 132.1, 131.5, 130.5, 124.3, 124.2, 120.6, 120.3, 116.2, 103.5, 63.9, 16.3, 14.9. HRMS (ESI+) calcd for C_18_H_18_NO_2_+ (M + H^+^): 280.1332, found: 280.1333.

#### 3.2.55. Synthesis of 5-Bromo-2-isopropylphenol (**41**-**a**)

To a solution of 4-bromo-1-isopropyl-2-methoxybenzene (100 mg, 0.44 mmol) in dry DCM (10 mL) at 0 °C was added BBr_3_ (133 mg, 0.53 mmol), and then the reaction was stirred at room temperature for 5 h. Upon completion of the reaction, the mixture was poured into ice-cold water (10 mL), and the volatiles were removed in vacuo. The resulting mixture was then diluted with EtOAc (20 mL) and water (10 mL). The organic layer was separated and washed with brine (10 mL × 2), dried over Na_2_SO_4_, filtrated, and evaporated under reduced pressure. The residue was purified by flash chromatography on silica gel (0–30% EA in PE) to give (**41**-**a**) as a white solid (43 mg, 45%). ^1^H NMR (400 MHz, DMSO-d_6_) δ 9.76 (d, *J* = 1.4 Hz, 1H), 7.04 (dd, *J* = 8.1, 1.4 Hz, 1H), 6.98–6.86 (m, 2H), 3.21–3.00 (m, 1H), 1.13 (d, *J* = 1.4 Hz, 6H). ESI-MS: *m*/*z* = 214.0 [M + H]^+^.

#### 3.2.56. Synthesis of 2-Isopropyl-5-(4,4,5,5-tetramethyl-1,3,2-dioxaborolan-2-yl)phenol (**41**-**b**)

To a solution of (**41**-**a**) (43 mg, 0.2 mmol) in 1,4-dioxane (10 mL) at room temperature under N_2_ atmosphere were added 4,4,4′,4′,5,5,5′,5′-octamethyl-2,2′-bi(1,3,2-dioxaborolane) (61 mg, 0.24 mmol), Pd(dppf)Cl_2_ (15 mg, 0.02 mmol), potassium acetate (39 mg, 0.4 mmol). After stirring at 85 °C for 12 h, the reaction mixture was cooled to room temperature, and the volatiles were removed in vacuo. The resulting mixture was then diluted with EtOAc (20 mL) and water (10 mL). The organic layer was separated and washed with brine (10 mL × 2), dried over Na_2_SO_4_, filtrated, and evaporated under reduced pressure. The residue was purified by flash chromatography on silica gel (0–30% EA in PE) to give **41**-**b** as a white solid (34 mg, 65%). ^1^H NMR (400 MHz, DMSO-d_6_) δ 9.19 (s, 1H), 7.14–7.05 (m, 3H), 3.26–3.12 (m, 1H), 1.26 (s, 12H), 1.14 (d, *J* = 6.8 Hz, 6H). ESI-MS: *m*/*z* = 285.2 [M + Na]^+^.

#### 3.2.57. 5-(6-Ethoxyisoquinolin-4-yl)-2-isopropylphenol (**41**)

Method A. The intermediate (**5**-**1**) was reacted with (**41**-**b**) through Suzuki coupling reaction to afford **41** as a white solid (60%). ^1^H NMR (400 MHz, DMSO-d_6_) δ 9.52 (s, 1H), 9.14 (s, 1H), 8.29 (s, 1H), 8.12 (d, *J* = 9.0 Hz, 1H), 7.36 (dd, *J* = 9.0, 2.3 Hz, 1H), 7.29 (d, *J* = 7.7 Hz, 1H), 7.24 (d, *J* = 2.3 Hz, 1H), 7.01–6.87 (m, 2H), 4.07 (q, *J* = 6.9 Hz, 2H), 3.28–3.22 (m, 1H), 1.36 (t, *J* = 6.9 Hz, 3H), 1.24 (d, *J* = 6.9 Hz, 6H). ESI-MS: *m*/*z* = 308.2 [M + H]^+^.

#### 3.2.58. Synthesis of (2,4-Dichloro-3-hydroxyphenyl)boronic Acid (**42**-**a**)

To a solution of (4-chloro-3-hydroxyphenyl)boronic acid (200 mg, 1.16 mmol) in acetonitrile (20 mL) was added N-chlorosuccinimide (186 mg, 1.39 mmol); the reaction was stirred at 80 °C for 12 h. Upon completion of the reaction, the volatiles were removed in vacuo. The resulting mixture was then diluted with EtOAc (30 mL) and water (15 mL). The organic layer was separated and washed with brine (10 mL × 2), dried over Na_2_SO_4_, filtrated, and evaporated under reduced pressure. The residue was purified by flash chromatography on silica gel (0–30% EA in PE) to give (**42**-**a**) as a yellow solid (65 mg, 27%). ^1^H NMR (400 MHz, DMSO-d_6_) δ 9.82 (s, 1H), 8.31 (s, 2H), 7.27 (d, *J* = 7.9 Hz, 1H), 6.84 (d, *J* = 7.9 Hz, 1H). ESI-MS: *m*/*z* = 207.0 [M + H]^+^.

#### 3.2.59. 2,6-Dichloro-3-(6-ethoxyisoquinolin-4-yl)phenol (**42**)

Method A. The intermediate (**5**-**1**) was reacted with (**42**-**a**) through Suzuki coupling reaction to afford **42** as a white solid (60%). ^1^H NMR (400 MHz, DMSO-d_6_) δ 9.20 (s, 1H), 8.25 (s, 1H), 8.13 (d, *J* = 9.0 Hz, 1H), 7.47 (d, *J* = 8.2 Hz, 1H), 7.34 (dd, *J* = 9.0, 2.5 Hz, 1H), 6.85 (d, *J* = 8.2 Hz, 1H), 6.66 (d, *J* = 2.5 Hz, 1H), 3.98 (q, *J* = 6.9 Hz, 2H), 1.32 (t, *J* = 6.9 Hz, 3H). ESI-MS: *m*/*z* = 334.0 [M + H]^+^.

#### 3.2.60. Synthesis of (2,4-Dichloro-5-hydroxyphenyl)boronic Acid (**43**-**a**)

To a solution of (4-chloro-3-hydroxyphenyl)boronic acid (200 mg, 1.16 mmol) in acetonitrile (20 mL) was added N-chlorosuccinimide (186 mg, 1.39 mmol); the reaction was stirred at 80 °C for 12 h. Upon completion of the reaction, the volatiles were removed in vacuo. The resulting mixture was then diluted with EtOAc (30 mL) and water (15 mL). The organic layer was separated and washed with brine (10 mL × 2), dried over Na_2_SO_4_, filtrated, and evaporated under reduced pressure. The residue was purified by flash chromatography on silica gel (0–30% EA in PE) to give (**43**-**a**) as a yellow solid (55 mg, 23%). ^1^H NMR (400 MHz, DMSO-d_6_) δ 10.28 (s, 1H), 8.31 (s, 2H), 7.34 (s, 1H), 6.97 (s, 1H). ESI-MS: *m*/*z* = 207.0 [M + H]^+^.

#### 3.2.61. 2,4-Dichloro-5-(6-ethoxyisoquinolin-4-yl)phenol (**43**)

Method A. The intermediate (**5**-**1**) was reacted with (**43**-**a**) through Suzuki coupling reaction to afford **43** as a white solid (55%). M.p. 198–200 °C. ^1^H NMR (400 MHz, DMSO-d_6_) δ 10.85 (s, 1H), 9.22 (s, 1H), 8.27 (s, 1H), 8.14 (d, *J* = 8.9 Hz, 1H), 7.69 (s, 1H), 7.35 (dd, *J* = 8.9, 2.4 Hz, 1H), 7.06 (s, 1H), 6.67 (d, *J* = 2.4 Hz, 1H), 4.02 (q, *J* = 7.0, 2H), 1.32 (t, *J* = 7.0 Hz, 3H). ^13^C NMR (126 MHz, DMSO) δ 160.7, 152.9, 152.3, 143.6, 135.7, 135.3, 130.6, 129.1, 124.0, 123.2, 121.1, 120.6, 119.9, 103.4, 64.08, 14.8. HRMS (ESI+) calcd for C_17_H_14_Cl_2_NO_2_+ (M + H^+^): 334.0396, found: 334.0397.

#### 3.2.62. Synthesis of (4-Chloro-2-fluoro-5-hydroxyphenyl)boronic Acid (**44**-**a**)

To a solution of (4-chloro-2-fluoro-5-methoxyphenyl)boronic acid (100 mg, 0.49 mmol) in dry DCM (10 mL) at 0 °C was added BBr_3_ (160 mg, 0.64 mmol); the reaction was stirred at room temperature for 5 h. Upon completion of the reaction, the mixture was poured into ice-cold water (10 mL), and the volatiles were removed in vacuo. The resulting mixture was then diluted with EtOAc (20 mL) and water (10 mL). The organic layer was separated and washed with brine (10 mL × 2), dried over Na_2_SO_4_, filtrated, and evaporated under reduced pressure. The residue was purified by flash chromatography on silica gel (0–30% EA in PE) to give (**44**-**a**) as a yellow solid (71 mg, 76%). ^1^H NMR (400 MHz, DMSO-d_6_) δ 9.94 (s, 1H), 8.19 (s, 2H), 7.15 (d, *J* = 8.4 Hz, 1H), 7.08 (d, *J* = 5.5 Hz, 1H). ESI-MS: *m*/*z* = 191.0 [M + H]^+^.

#### 3.2.63. 2-Chloro-5-(6-ethoxyisoquinolin-4-yl)-4-fluorophenol (**44**)

Method A. The intermediate (**5**-**1**) was reacted with (**44**-**a**) through Suzuki coupling reaction to afford **44** as a yellow solid (60%). ^1^H NMR (400 MHz, Chloroform-d) δ 9.10 (s, 1H), 8.44 (s, 1H), 7.93 (d, *J* = 9.1 Hz, 1H), 7.37–7.20 (m, 3H), 7.09 (d, *J* = 6.5 Hz, 1H), 6.91 (s, 1H), 4.06 (q, *J* = 7.0 Hz, 2H), 1.44 (t, *J* = 7.0 Hz, 3H). ESI-MS: *m*/*z* = 318.1 [M + H]^+^.

#### 3.2.64. 5-(6-Ethoxyisoquinolin-4-yl)-2-fluorophenol (**45**)

Method A. The intermediate (**5**-**1**) was reacted with (4-fluoro-3-hydroxyphenyl)boronic acid through Suzuki coupling reaction to afford **45** as a white solid (65%). M.p. 209–211 °C. ^1^H NMR (400 MHz, DMSO-d_6_) δ 10.12 (s, 1H), 9.16 (s, 1H), 8.29 (s, 1H), 8.12 (d, *J* = 9.0 Hz, 1H), 7.35 (dd, *J* = 9.0, 2.6 Hz, 1H), 7.31 (dd, ^3^*J*_F-H_ = 11.4 Hz, ^3^*J*_H-H_ = 8.4 Hz, 1H), 7.12 (d, *J* = 2.6 Hz, 1H), 7.09 (dd, ^4^*J*_F-H_ = 9.5 Hz, ^4^*J*_H-H_ = 2.2 Hz, 1H), 6.95 (ddd, ^3^*J*_H-H_ = 8.3 Hz, ^4^*J*_F-H_ = 4.3 Hz, ^4^*J*_H-H_ = 2.2 Hz, 1H), 4.06 (q, *J* = 6.9 Hz, 2H), 1.35 (t, *J* = 6.9 Hz, 3H). ^13^C NMR (126 MHz, DMSO) δ 160.2, 151.1, 150.8 (d, ^1^*J* _C-F_ = 240.3 Hz), 145.0 (d, ^2^*J* _C-F_ = 12.6 Hz), 142.8, 135.1, 133.2, 130.8, 130.1, 123.8, 120.7, 119.9, 119.0, 116.5 (d, ^2^*J* _C-F_ = 17.6 Hz), 102.9, 63.5, 14.4. HRMS (ESI+) calcd for C_17_H_15_FNO_2_+ (M + H^+^): 284.1081, found: 284.1081.

#### 3.2.65. 5-(6-Ethoxyisoquinolin-4-yl)-2,4-difluorophenol (**46**)

Method A. The intermediate (**5**-**1**) was reacted with (2,4-difluoro-5-hydroxyphenyl)boronic acid through Suzuki coupling reaction to afford **46** as a white solid (45%). M.p. 230–232 °C. ^1^H NMR (600 MHz, DMSO-d_6_) δ 9.21 (s, 1H), 8.32 (s, 1H), 8.28 (s, 1H), 8.14 (d, *J* = 9.0 Hz, 1H), 7.41 (dd, ^3^*J*_F-H_ = 11.2, 9.6 Hz, 1H), 7.36 (dd, *J* = 9.0, 2.4 Hz, 1H), 7.03 (dd, ^4^*J*_F-H_ = 9.7, 7.3 Hz, 1H), 6.83 (d, *J* = 2.5 Hz, 1H), 4.05 (q, *J* = 6.9 Hz, 2H), 1.34 (t, *J* = 6.9 Hz, 3H). ^13^C NMR (151 MHz, DMSO) δ 160.3, 151.6 (dd, ^1^*J*_C-F_ = 132.3 Hz, ^2^*J*_C-F_ = 10.1 Hz), 151.8, 150.5 (dd, ^1^*J*_C-F_ = 205.4 Hz, ^2^*J*_C-F_ = 10.1 Hz), 143.7, 141.9 (dd, ^2^*J* _C-F_ = 12.2 Hz, ^3^*J*_C-F_ = 2.8 Hz), 135.5, 130.2, 125.4, 123.6, 120.1, 119.7 (dd, ^2^*J* = 17.7 Hz, ^3^*J* = 3.4 Hz), 119.5, 105.1 (dd, ^2^*J*_C-F_ = 22.7 Hz, ^3^*J*_C-F_ = 18.9 Hz), 102.9, 63.6, 14.4. HRMS (ESI+) calcd for C_17_H_14_F_2_NO_2_+ (M + H^+^): 302.0987, found: 302.0986. HPLC analysis: 14.260 min, 99.5% purity.

#### 3.2.66. 4-(6-Ethoxyisoquinolin-4-yl)pyridin-2(1H)-one (**47**)

Method A. The intermediate (**5**-**1**) was reacted with (2-oxo-1,2-dihydropyridin-4-yl)boronic acid through Suzuki coupling reaction to afford **47** as a white solid (50%). ^1^H NMR (400 MHz, DMSO-d_6_) δ 11.79 (s, 1H), 9.21 (s, 1H), 8.35 (s, 1H), 8.14 (d, *J* = 9.0 Hz, 1H), 7.51 (d, *J* = 6.6 Hz, 1H), 7.38 (dd, *J* = 9.0, 2.4 Hz, 1H), 7.14 (d, *J* = 2.4 Hz, 1H), 6.45 (d, *J* = 1.7 Hz, 1H), 6.37 (dd, *J* = 6.6, 1.7 Hz, 1H), 4.12 (q, *J* = 6.9 Hz, 2H), 1.37 (t, *J* = 6.9 Hz, 3H). ESI-MS: *m*/*z* = 267.1 [M + H]^+^.

#### 3.2.67. 5-(6-Ethoxyisoquinolin-4-yl)pyridin-2(1H)-one (**48**)

Method A. The intermediate (**5**-**1**) was reacted with (6-oxo-1,6-dihydropyridin-3-yl)boronic acid through Suzuki coupling reaction to afford **48** as a white solid (55%). ^1^H NMR (400 MHz, DMSO-d_6_) δ 11.90 (s, 1H), 9.14 (s, 1H), 8.31 (s, 1H), 8.11 (d, *J* = 9.0 Hz, 1H), 7.66 (dd, *J* = 9.4, 2.7 Hz, 1H), 7.58 (d, *J* = 2.4 Hz, 1H), 7.35 (dd, *J* = 9.0, 2.4 Hz, 1H), 7.06 (d, *J* = 2.4 Hz, 1H), 6.49 (dd, *J* = 9.4, 0.7 Hz, 1H), 4.12 (q, *J* = 7.0 Hz, 2H), 1.37 (t, *J* = 7.0 Hz, 3H). ESI-MS: *m*/*z* = 267.1 [M + H]^+^.

### 3.3. Cell Lines and Cell Culture

The human prostate cancer cell lines LASCPC-01 and PC-3 were obtained from the American Type Culture Collection (ATCC). HITES medium modified with hydrocortisone, human recombinant insulin, transferrin, and estradiol was used to culture LASCPC-01 cells. PC-3 cells were cultured in F12 medium (Corning Cellgro, Manassas, VA, USA), and 10% fetal bovine serum (Gibco, Grand Island, NY, USA) and 100 U/mL penicillin–streptomycin were added to all tumor cell complete media. All the cells were cultured in a humidified incubator at 37 °C with 5% CO_2_.

### 3.4. Determination of In Vitro Anticancer Activity

The overall growth of human cancer cell lines was determined using the CCK-8 assay. Briefly, cells were seeded into 96-microwell plates at an appropriate density (6000 cells/well for LASCPC-01 cell line, 3000 cells/well for PC-3 cell line), with 100 μL of complete medium per well, and cultured overnight. Various concentrations of the compounds were added, with three replicate wells for each concentration. The cells were incubated at 37 °C with 5% CO_2_ for 72 h. Subsequently, 10 μL of CCK-8 reagent was added to each well, and the plates were incubated in a 37 °C incubator for 2–4 h. The optical density (OD) at 450 nm was measured using a SpectraMax 190 microplate reader. The inhibition rate of cell proliferation by the compound was calculated using the following formula: Inhibition rate (%) = [1 − (OD_drug − OD_negative control)/(OD_positive control − OD_negative control)] × 100%. The IC_50_ value was determined using the four-parameter logistic regression model. Each experiment was independently repeated three times, with three replicate wells for each concentration in each experiment. Results are presented as mean ± SD.

### 3.5. Flow Cytometry Analysis for Cell Cycle

Cells were seeded into 12-well plates at an appropriate density and cultured overnight. Various concentrations of the compounds were added. The cells were incubated at 37 °C with 5% CO_2_ for 24 h. Then, cells were collected and washed with PBS, then fixed with 70% ethanol and stored at −20 °C overnight. After centrifugation at 300× *g* for 5 min, the cells were then incubated with RNaseA (Sangon Biotech, Shanghai, China) and stained with propidium iodide (Beyotime, Haimen, China) for 15 min at room temperature. The cells were examined using a FACS Calibur flow cytometer (BD Biosciences, Dubai, UAE). Data were analyzed in FlowJo software (FlowJo 7.6, Becton Dickinson & Co., Hong Kong). Each experiment was performed in triplicate.

### 3.6. Western Blot Analysis

The cells were inoculated in six-well plates and cultured overnight, treated with different concentrations of compounds for 24 h, and then collected. The cells were washed with PBS once, and the cells were lysed with 1 × SDS loading buffer (50 mM Tris pH 6.8, 100 mM DTT, 2% SDS, 0.1% bromophenol blue, 10% glycerol). The cell lysates were collected, heated in a boiling water bath for 10 min, and centrifuged at 4 °C 12,000 rpm for 5 min. The supernatant was taken for SDS-PAGE electrophoresis. After electrophoresis, the proteins were transferred to the NC membrane using a fast wet rotation apparatus. After the transfer, the target strips were sealed with a sealing solution containing 5% skimmed milk powder (5% skimmed milk powder, 20 mM Tris-HCl, pH 7.2–7.4, 150 mM NaCl, 0.1% Tween-20) at room temperature on a shaker for 1 h. The membrane was then incubated overnight in primary antibody at 4 °C and washed with TBST washing solution (100 mM Tris-HCl pH 7.2–7.4, 0.9% NaCl, 0.2% Tween-20) at room temperature three times, 10 min each time, and then subsequently with secondary horseradish peroxidase (HRP)-conjugated goat anti-Mouse or anti-rabbit IgG antibody. Finally, immunoreactive proteins were captured with Image Quant LAS4000 (GE Healthcare, Chicago, IL, USA).

### 3.7. Antibodies

DDC (#13561), Cleaved PARP (#5625S), Cleaved Caspase-3 (#9664), Caspase-3 (#9665S) were all purchased from Cell Signaling Technology (CST, Danvers, MA, USA). Additional antibodies included Anti-Chromogranin A (#ab283265, Abcam, Cambridge, UK); Anti-Synaptophysin (#ab32127, Abcam); NCAM1(#14255-1-AP, Proteintech, Rosemont, IL, USA); GAPDH (#60004-1, Proteintech); β-Actin (#HRP-60008, Proteintech); Goat Anti-Mouse IgG (#401215, Calbiochem, San Diego, CA, USA); Goat Anti-Rabbit IgG (#401353, Calbiochem).

## 4. Conclusions

In summary, by simplifying the scaffold of the natural product lycobetaine, we obtained the active compound **1**, characterized by a 4-position aryl-substituted isoquinoline, which exhibited obvious in vitro antiproliferative activity against the NEPC cell line LASCPC-01. Further structural optimization and systematic SAR studies on compound **1** led to the discovery of compound **46**, which exhibited potent antiproliferative activity against the LASCPC-01 cell line, as well as excellent selectivity over prostate cancer cell line PC-3, with a selectivity index greater than 190-fold, though it is noted that there are inherent differences between the NEPC cell line LASCPC-01 and the non-NPEC cell line PC-3. These cell line-specific alterations can also lead to selectivity. Therefore, we will further validate our results using different in vitro models. Additionally, compound **46**, along with the natural products lycobetaine and sanguinarine, could induce G1 cell cycle arrest and apoptosis. However, compound **46** and the two natural products showed completely different effects on the regulation of neuroendocrine-related proteins, suggesting that its anti-cancer activity may be mediated through different mechanisms, which warrants further investigation.

## Figures and Tables

**Table 1:** Antiproliferative activities of the two natural products and carbidopa against LASCPC-01 and PC-3 cell lines.

Compd.	LASCPC-01 *^a^* IC_50_ (μM)	PC-3 *^a^* IC_50_ (μM)	SI *^b^*	*p* Value *^c^*
Carbidopa	98.82 ± 10.17	160.35 ± 11.08	1.6	***
Sanguinarine	0.96 ± 0.64	1.92 ± 0.55	2.0	ns
Lycobetaine	0.34 ± 0.16	3.85 ± 0.21	11.3	***

*^a^* IC_50_ values are the mean of three independent experiments. *^b^* SI (selectivity index) was calculated by the formula SI = IC_50_ (PC-3)/IC_50_ (LASCPC-01). *^c^ p* > 0.05, ns; *p* ≤ 0.001, ***.

**Table 2:** Antiproliferative activities of compounds **1**–**8** against LASCPC-01 cell line.

Compd.	Structure	LASCPC-01 *^a^* IC_50_ (μM)
**Carbidopa**	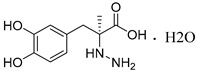	98.82 ± 10.17
**1**	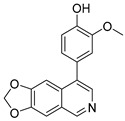	13.79 ± 1.87
**2**	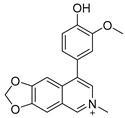	>90
**3**	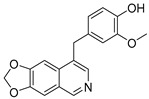	29.54 ± 10.46
**4**	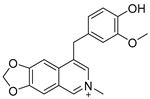	>90
**5**	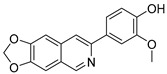	>90
**6**	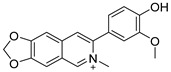	>90
**7**	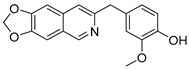	>90
**8**	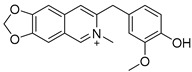	45.23 ± 5.31

*^a^* IC_50_ values are the mean of three independent experiments.

**Table 3:** Antiproliferative activities of compounds **9**–**21** against LASCPC-01 cell line.

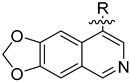					
Compd.	R	LASCPC-01 *^a^* IC_50_ (μM)	Compd.	R	LASCPC-01 *^a^* IC_50_ (μM)
**Carbidopa**	-	98.82 ± 10.17	**15**	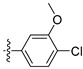	85.90 ± 5.53
**1**	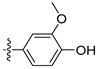	9.27 ± 0.48	**16**	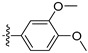	39.98 ± 12.03
**9**	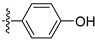	>90	**17**	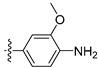	49.85 ± 13.40
**10**	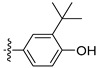	30.70 ± 1.14	**18**	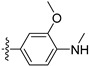	30.43 ± 6.17
**11**	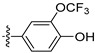	31.17 ± 1.50	**19**	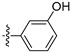	7.48 ± 3.41
**12**	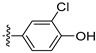	76.87 ± 6.04	**20**	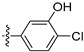	9.96 ± 0.64
**13**	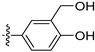	56.54 ± 8.05	**21**	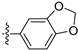	>90
**14**	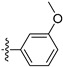	70.45 ± 29.68			

*^a^* IC_50_ values are the mean of three independent experiments.

**Table 4:** Antiproliferative activities of compounds **22**–**36** against LASCPC-01 cell line.

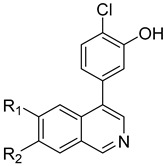							
Compd.	R_1_	R_2_	LASCPC-01 *^a^* IC_50_ (μM)	Compd.	R_1_	R_2_	LASCPC-01 *^a^* IC_50_ (μM)
**Carbidopa**	-		98.82 ± 10.17	**29**	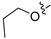	H	64.46 ± 15.51
**20**	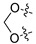		9.96 ± 0.64	**30**	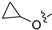	H	>90
**22**	H	H	34.73 ± 15.85	**31**	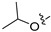	H	>90
**23**	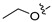	H	6.90 ± 1.95	**32**	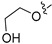	H	5.09 ± 2.96
**24**	H	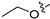	>90	**33**	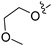	H	10.98 ± 1.56
**25**	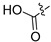	H	>90	**34**	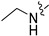	H	4.47 ± 0.9
**26**	H	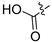	51.42 ± 32.29	**35**	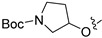	H	7.47 ± 1.46
**27**	H	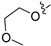	35.94 ± 9.82	**36**	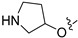	H	13.23 ± 1.03
**28**	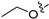	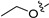	12.92 ± 3.14				

*^a^* IC_50_ values are the mean of three independent experiments.

**Table 5:** Antiproliferative activities of compounds **37**–**48** against LASCPC-01 cell line.

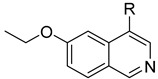					
Compd.	R	LASCPC-01 *^a^* IC_50_ (μM)	Compd.	R	LASCPC-01 *^a^* IC_50_ (μM)
**Carbidopa**	-	98.82 ± 10.17	**42**	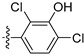	22.43 ± 4.04
**23**	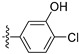	6.90 ± 1.95	**43**	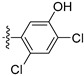	3.41 ± 0.04
**37**	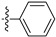	10.43 ± 3.42	**44**	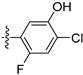	30.5 ± 1.63
**38**	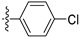	6.42 ± 4.15	**45**	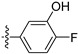	1.2 ± 0.03
**39**	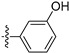	1.78 ± 0.20	**46**	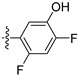	0.47 ± 0.14
**40**	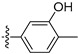	5.06 ± 0.37	**47**	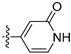	13.85 ± 2.18
**41**	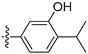	>90	**48**	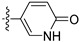	44.21 ± 18.36

*^a^* IC_50_ values are the mean of three independent experiments.

**Table 6:** Antiproliferative activities of representative compounds against LASCPC-01 and PC-3 cell lines.

Compd.	LASCPC-01 *^a^* IC_50_ (μM)	PC-3 *^a^* IC_50_ (μM)	SI *^b^*	*p* Value *^c^*
**1**	9.27 ± 0.48	>90	>9.7	***
**20**	9.96 ± 0.64	>90	>9.0	***
**23**	3.71 ± 1.99	>90	>24.3	***
**28**	12.92 ± 3.14	24.85 ± 5.25	2.3	ns
**32**	5.09 ± 2.96	>90	>17.7	***
**33**	10.98 ± 1.56	>90	>8.2	***
**34**	4.47 ± 0.9	>90	>20.1	***
**35**	7.47 ± 1.46	16.47 ± 3.30	2.2	ns
**36**	13.23 ± 1.03	39.35 ± 4.95	3.0	***
**37**	10.43 ± 3.42	74.11 ± 11.68	7.1	***
**38**	3.93 ± 0.22	33.32 ± 2.99	8.5	**
**39**	1.78 ± 0.20	>90	>50.6	***
**40**	5.06 ± 0.37	>90	>17.8	***
**43**	3.41 ± 0.04	>90	>26.4	***
**45**	1.20 ± 0.03	>90	>75	***
**46**	0.47 ± 0.14	>90	>191.5	***
**47**	13.85 ± 2.18	>90	>6.5	***

*^a^* IC_50_ values are the mean of three independent experiments. *^b^* SI (selectivity index) was calculated by the formula SI = IC_50_ (PC-3)/IC_50_ (LASCPC-01). *^c^ p* > 0.05, ns; *p* ≤ 0.001, ***.

## Data Availability

Data are contained within the article and Supplementary Materials.

## Supplementary Materials

The following supporting information can be downloaded at: https://www.mdpi.com/article/10.3390/molecules29184503/s1. ^1^H and ^13^C NMR and HPLC spectra of representative compounds.

## Abbreviations

ADT, androgen deprivation therapy; AR, androgen receptor; DCM, Dichloromethane; DDC, DOPA decarboxylase; DMF, N,N-Dimethylformamide; DOPA, Dihydroxyphenylalanine; EA, Ethyl acetate; EZH2, Enhancer of zeste homolog 2; NE, neuroendocrine; NEPC, neuroendocrine prostate cancer; PC, prostate cancer; PE, petroleum ether; PSA, prostate-specific antigen; SAR, structure–activity relationship; TBDMSCl, *tert*-Butyldimethylsilyl chloride; rt, room temperature; TFA, Trifluoroacetic acid.
