# Supplementary material for: Structural Optimization of Isoquinoline Derivatives from Lycobetaine and Their Inhibitory Activity against Neuroendocrine Prostate Cancer Cells
Source: Molecules. 2024 Sep 23;29(18):4503. doi: 10.3390/molecules29184503 (PMC11435415; doi:10.3390/molecules29184503)

Supplementary Information

Structural Optimization of Isoquinoline Derivatives from Lycobetaine and Their Inhibitory Activity against Neuroendocrine Prostate Cancer Cells

Zhuo Zhang [1,†], Yiyi Ji [2,†], Qianqian Shen [3], Yanjie Ma [4,5], Haiyang Hou [3,6], Huajie Yang [4], Yinjie Zhu [2,*], Yi Chen [3,5] and Youhong Hu [1,4,5,6,7,*]

[1] School of Chinese Materia Medica, College of Pharmacy, Nanjing University of Chinese Medicine, No. 138 Xianlin Road, Nanjing 210023, China; s18-zhangzhuo@simm.ac.cn
[2] Department of Urology, Renji Hospital, Shanghai Jiao Tong University School of Medicine, Shanghai 200127, China; jiyiyi@sjtu.edu.cn
[3] State Key Laboratory of Chemical Biology, Shanghai Institute of Materia Medica, Chinese Academy of Sciences, Shanghai 201203, China; shenqianqian@simm.ac.cn (Q.S.); houhaiyang@simm.ac.cn (H.H.)
[4] State Key Laboratory of Drug Research, Shanghai Institute of Materia Medica, Chinese Academy of Sciences, 555 Zu-ChongZhi Road, Shanghai 201203, China; myj1007@simm.ac.cn (Y.M.); yanghuajie@simm.ac.cn (H.Y.)
[5] Shandong Laboratory of Yantai Drug Discovery, Bohai Rim Advanced Research Institute for Drug Discovery, Yantai 264117, China
[6] University of Chinese Academy of Sciences, No. 19A Yuquan Road, Beijing 100049, China
[7] School of Pharmaceutical Science and Technology, Hangzhou Institute for Advanced Study, University of Chinese Academy of Sciences, 1st Xiangshan Branch Alley, Hangzhou 310024, China
* Correspondence: yinjiezhu@outlook.com (Y.Z.); yhhu@simm.ac.cn (Y.H.)
† These authors contributed equally to this work.

Supporting Information

Content

1. NMR Spectrums of representative compounds···················S3–S13

2. HPLC Spectrums of representative compounds············· ·S14–S15

1. NMR Spectrums

^{1}H NMR Spectrum of compound **1**

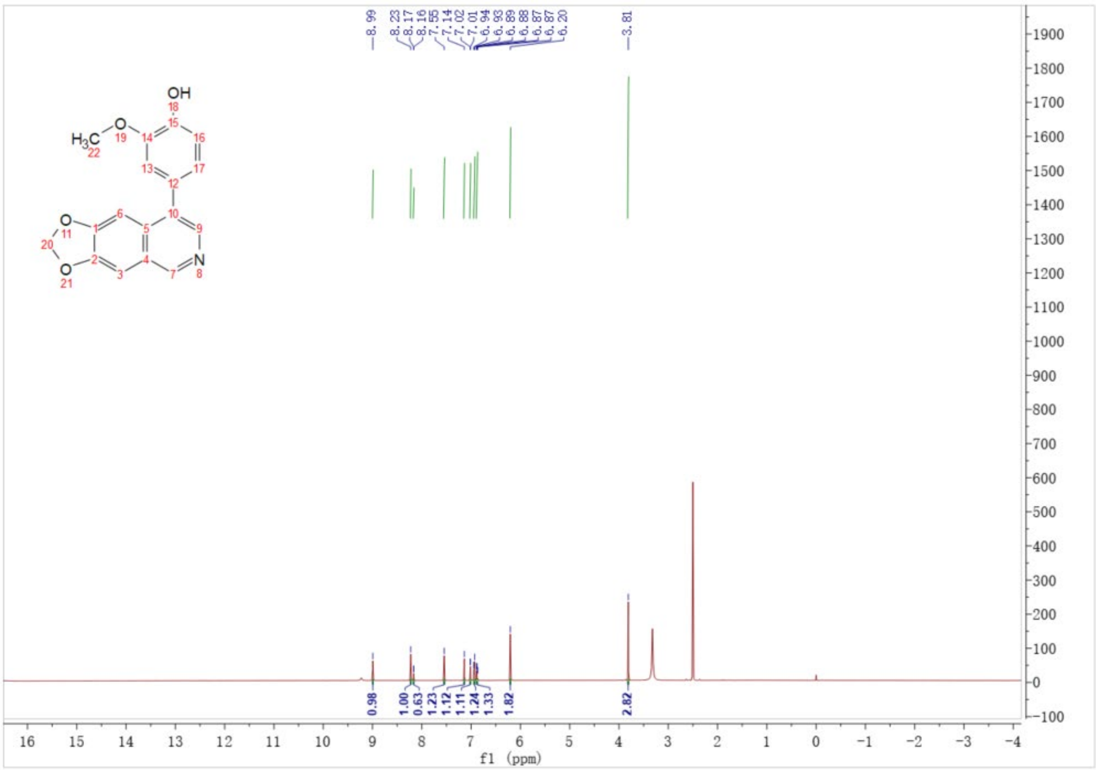

^{13}C NMR Spectrum of compound **1**

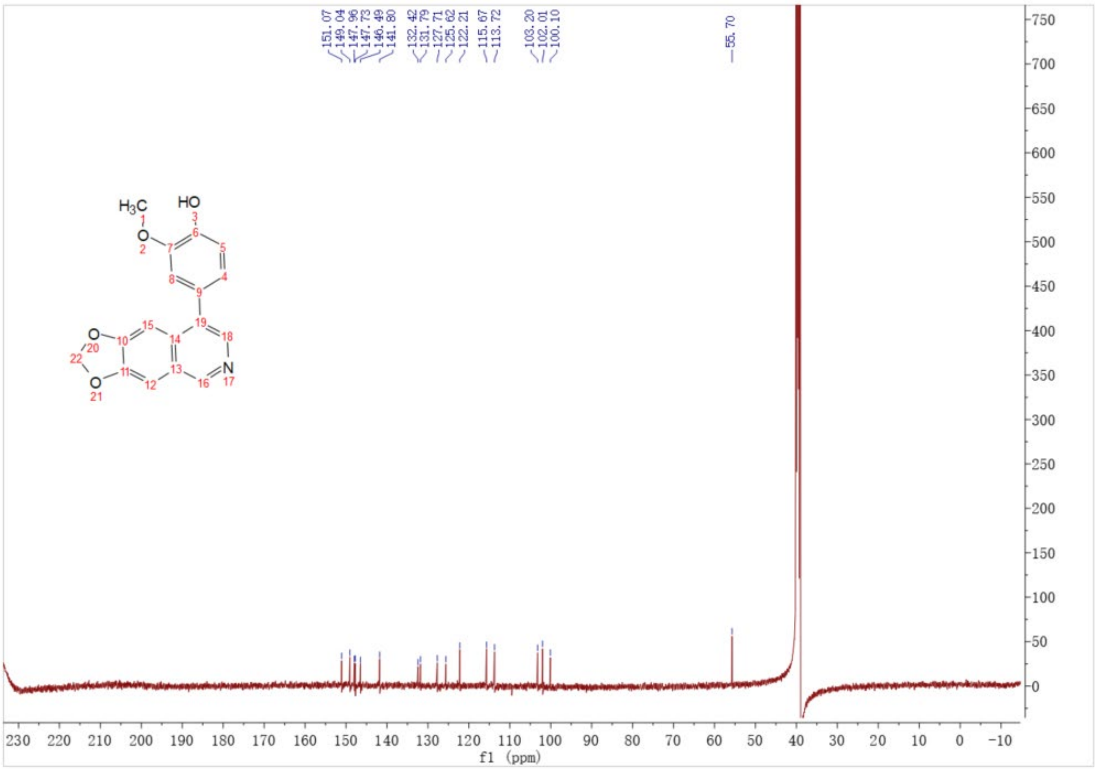

^{1}H NMR Spectrum of compound **19**

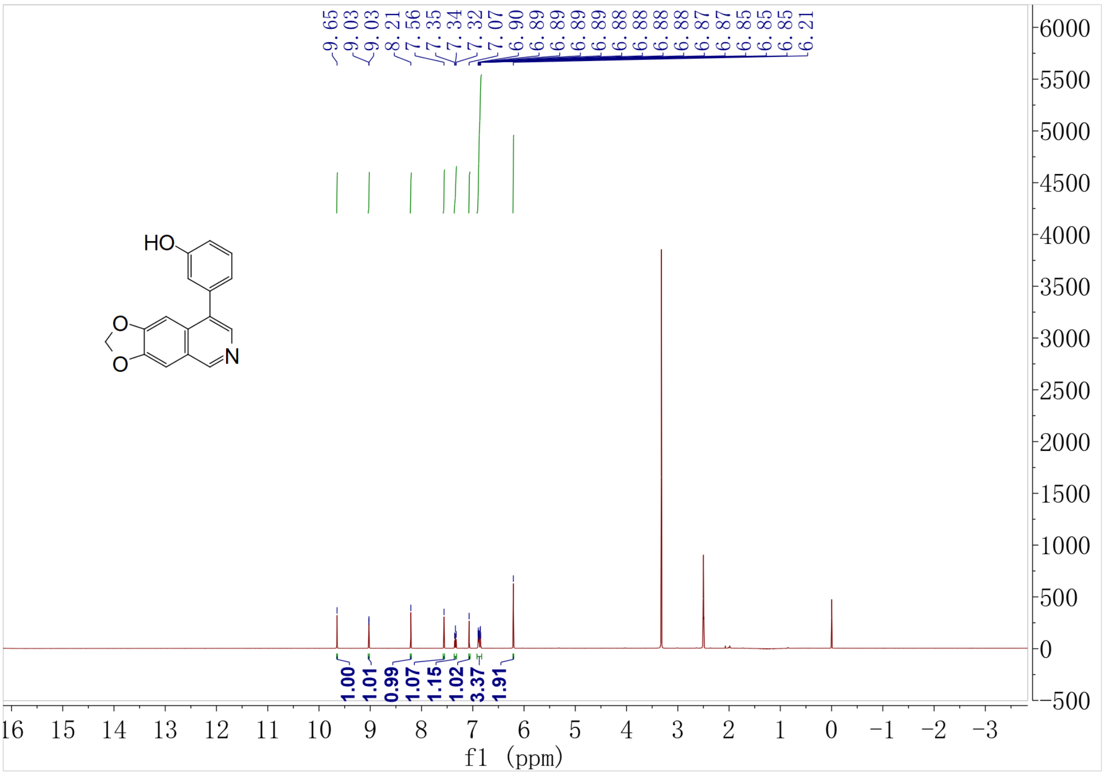

^{13}C NMR Spectrum of compound **19**

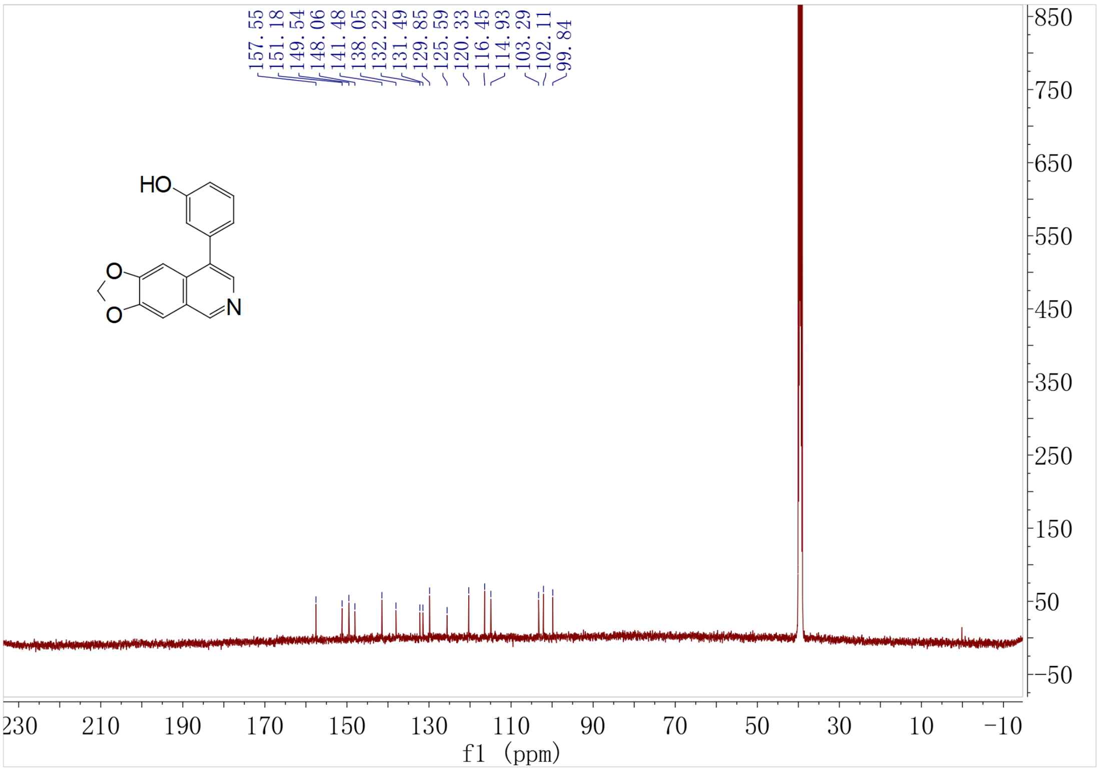

^{1}H NMR Spectrum of compound **20**

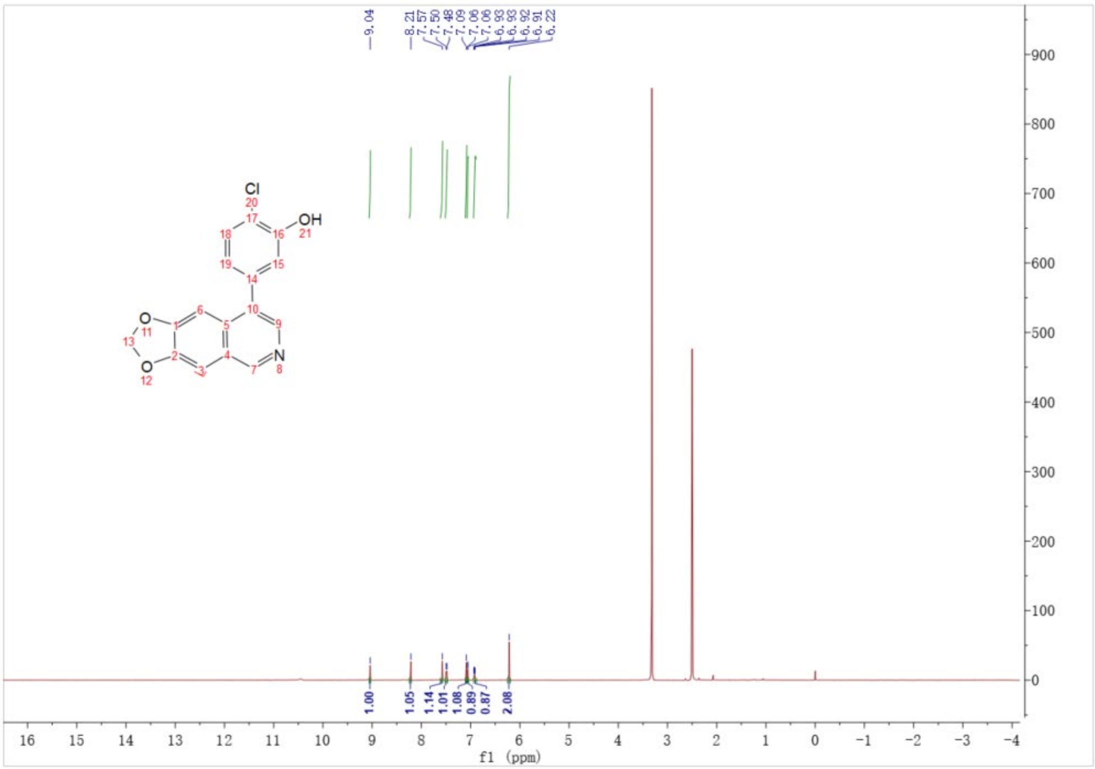

^{13}C NMR Spectrum of compound **20**

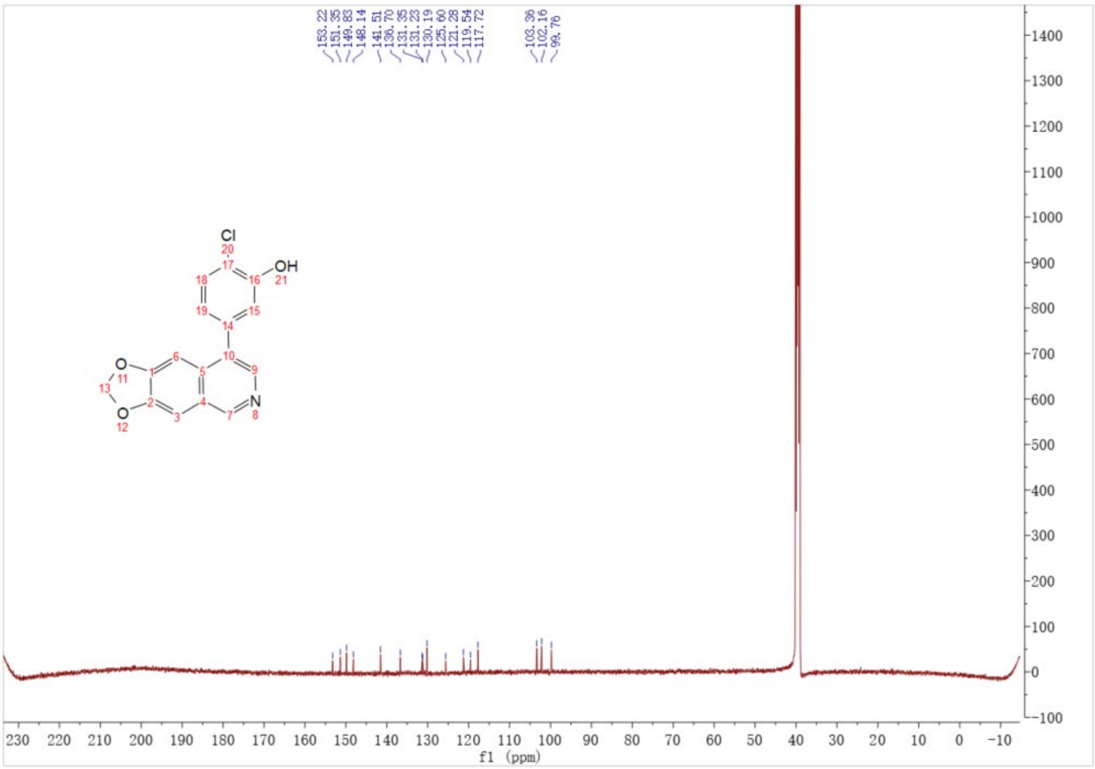

^{1}H NMR Spectrum of compound **23**

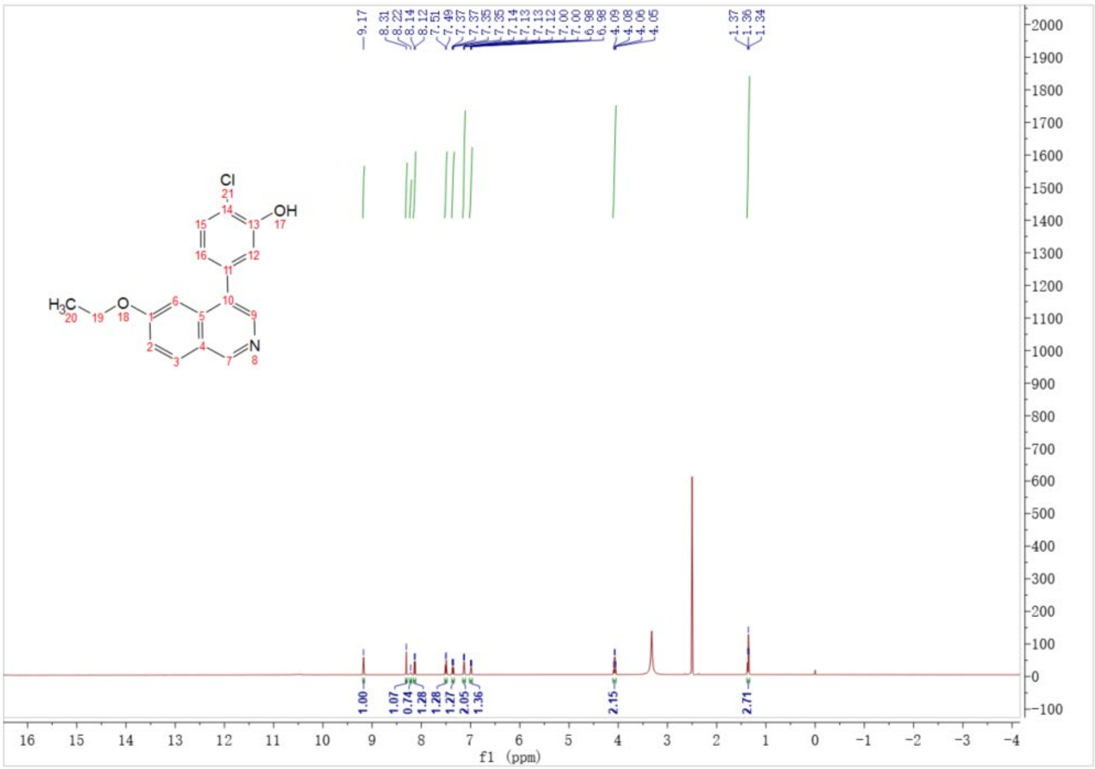

^{13}C NMR Spectrum of compound **23**

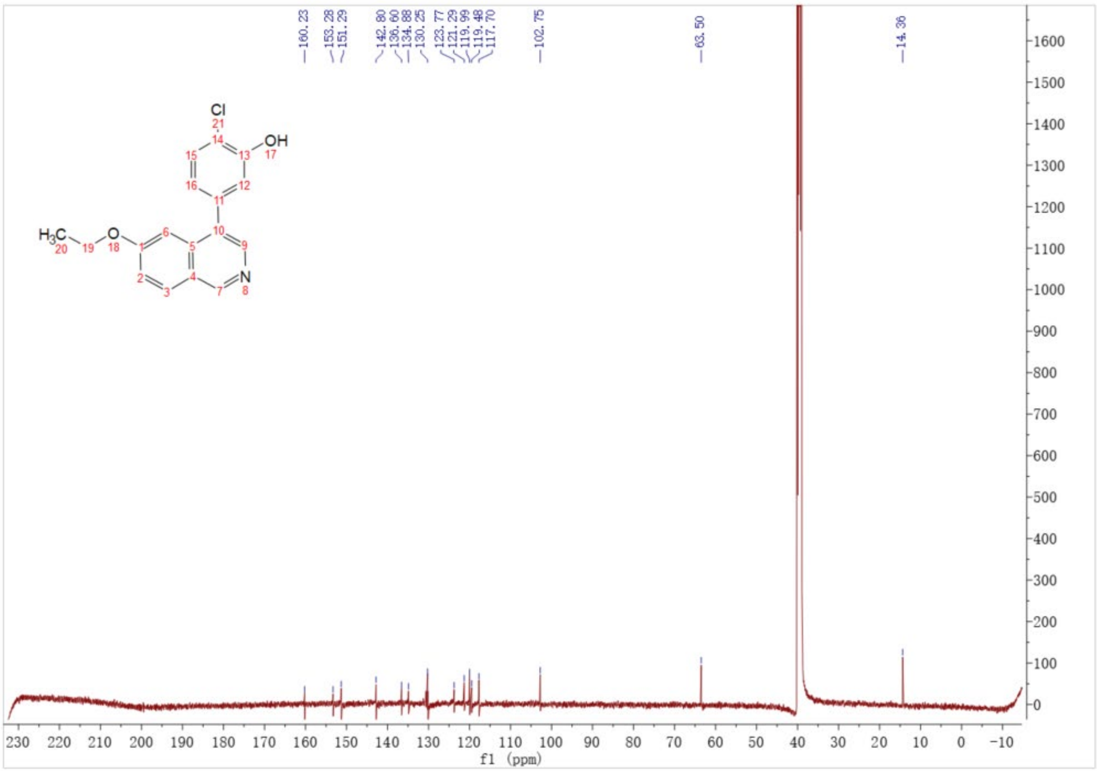

^{1}H NMR Spectrum of compound **34**

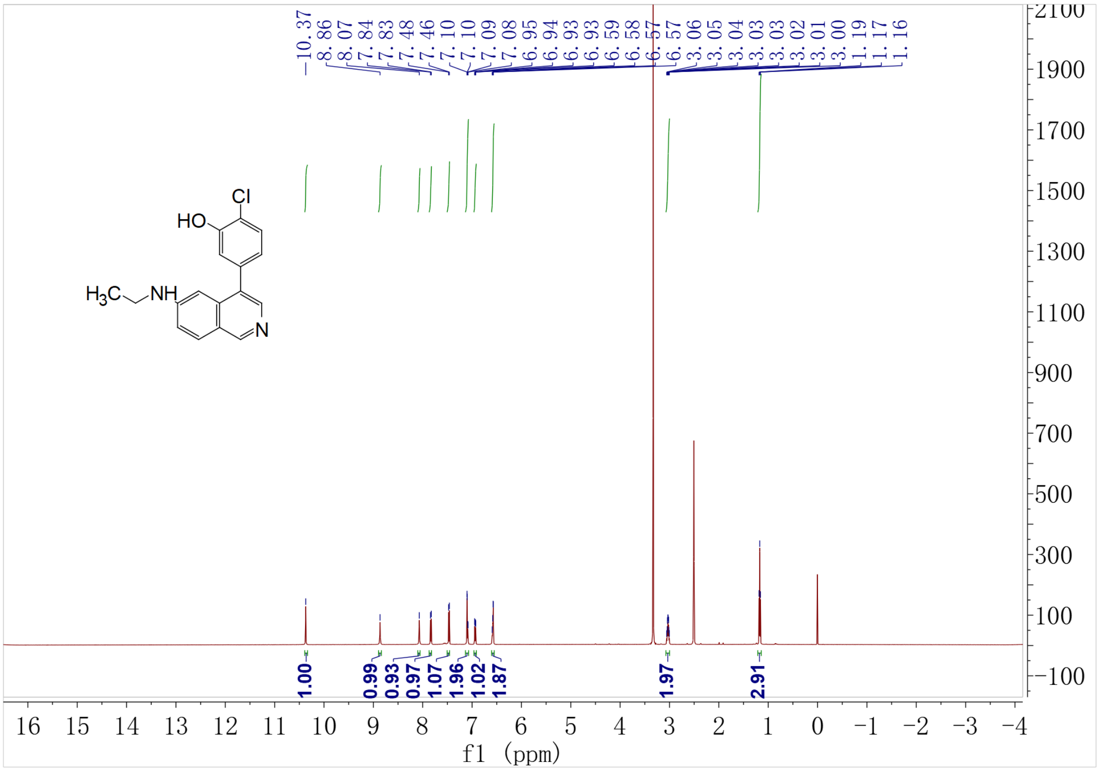

^{13}C NMR Spectrum of compound **34**

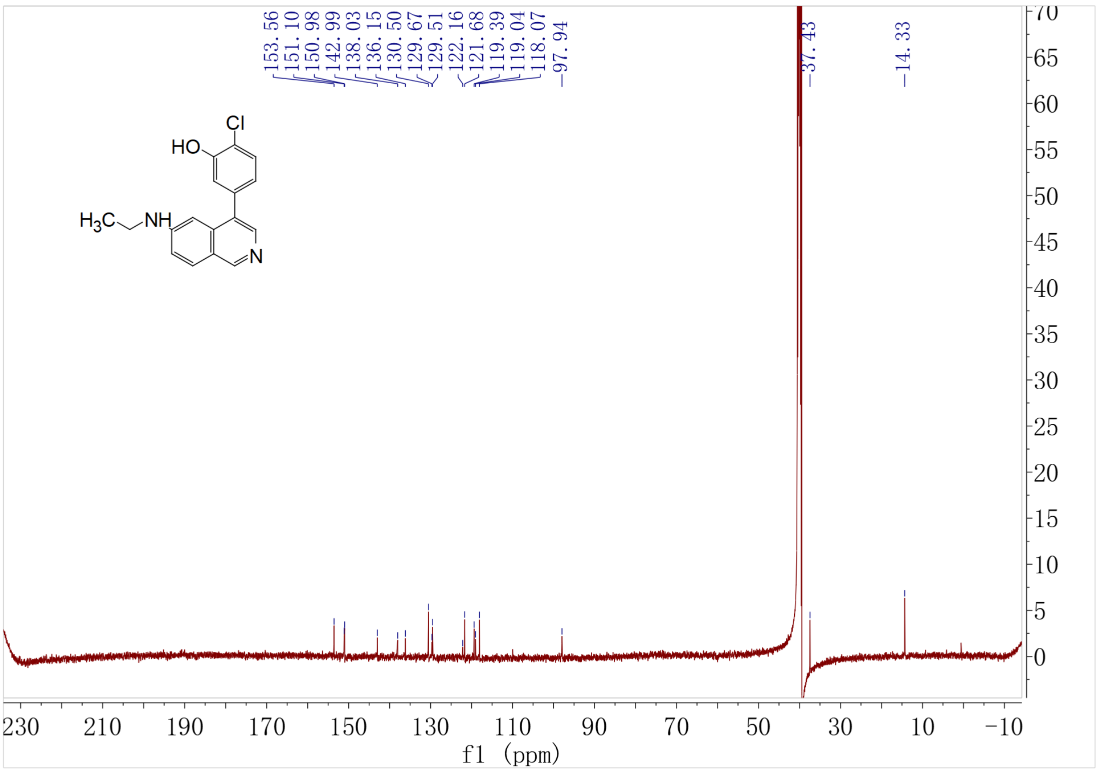

^{1}H NMR Spectrum of compound **37**

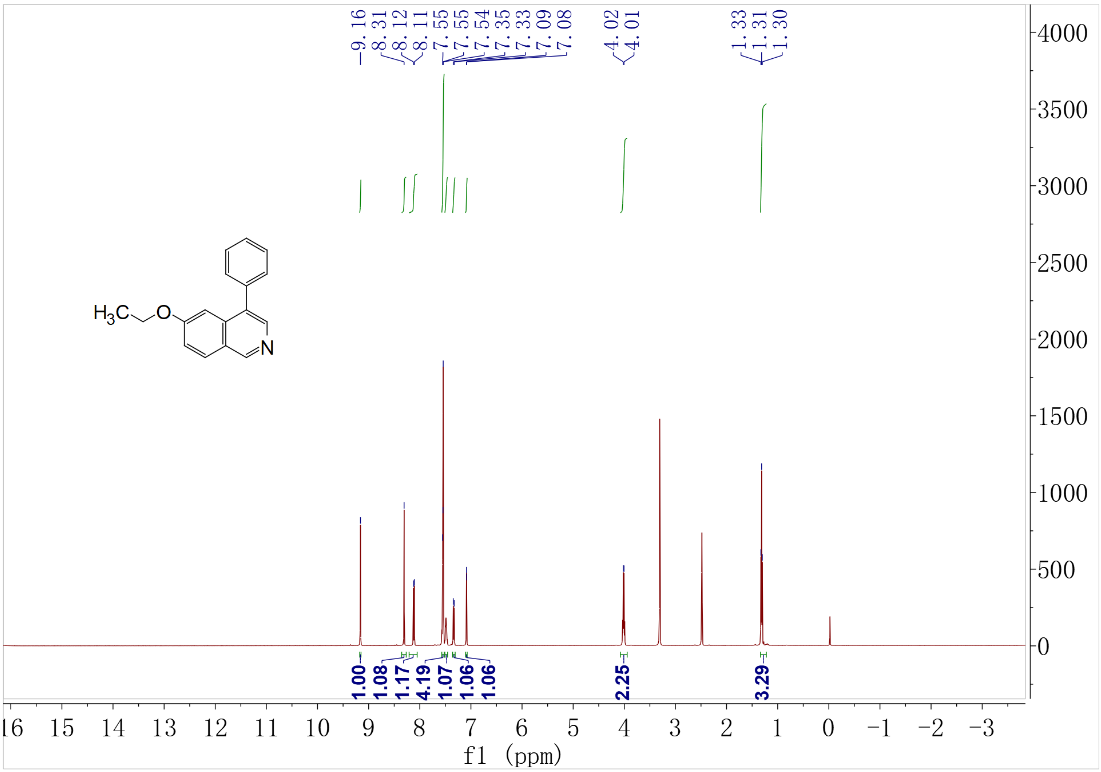

^{13}C NMR Spectrum of compound **37**

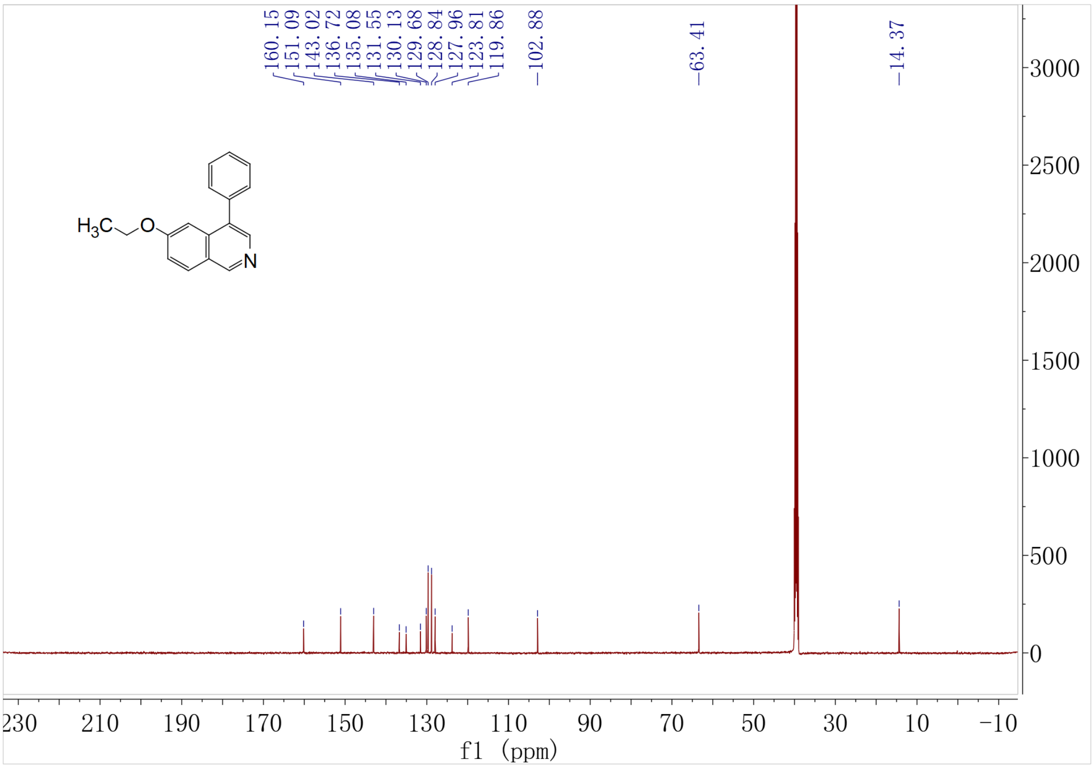

^{1}H NMR Spectrum of compound **38**

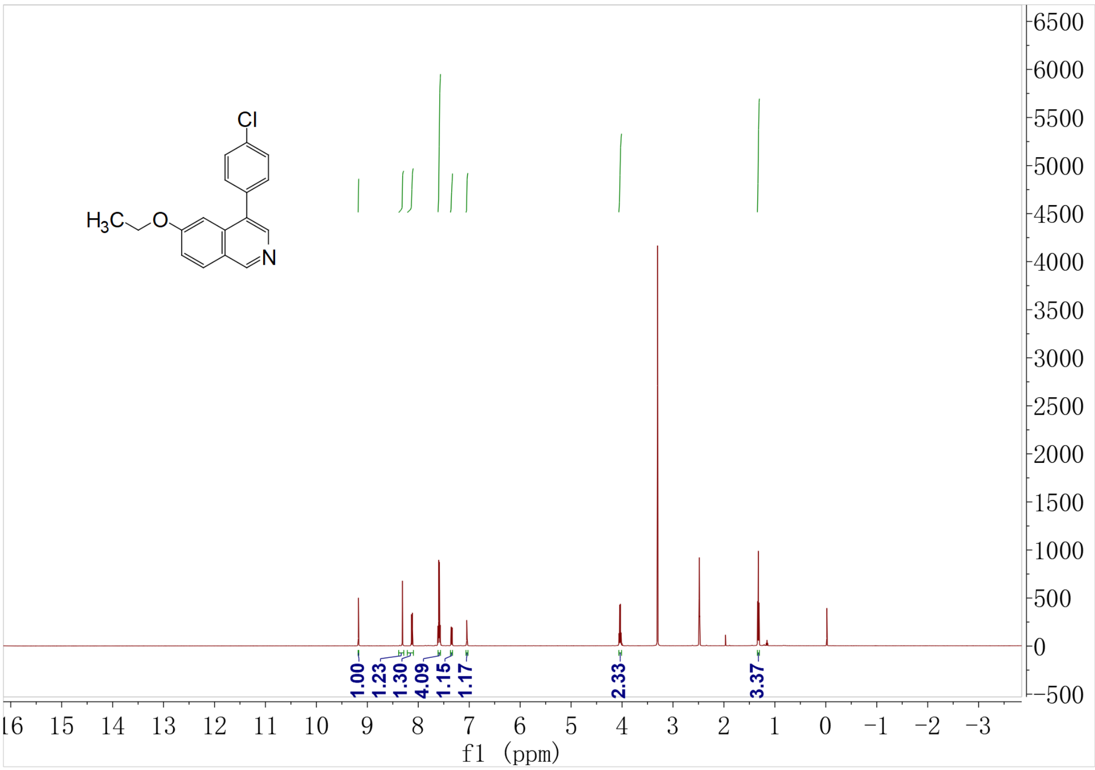

^{13}C NMR Spectrum of compound **38**

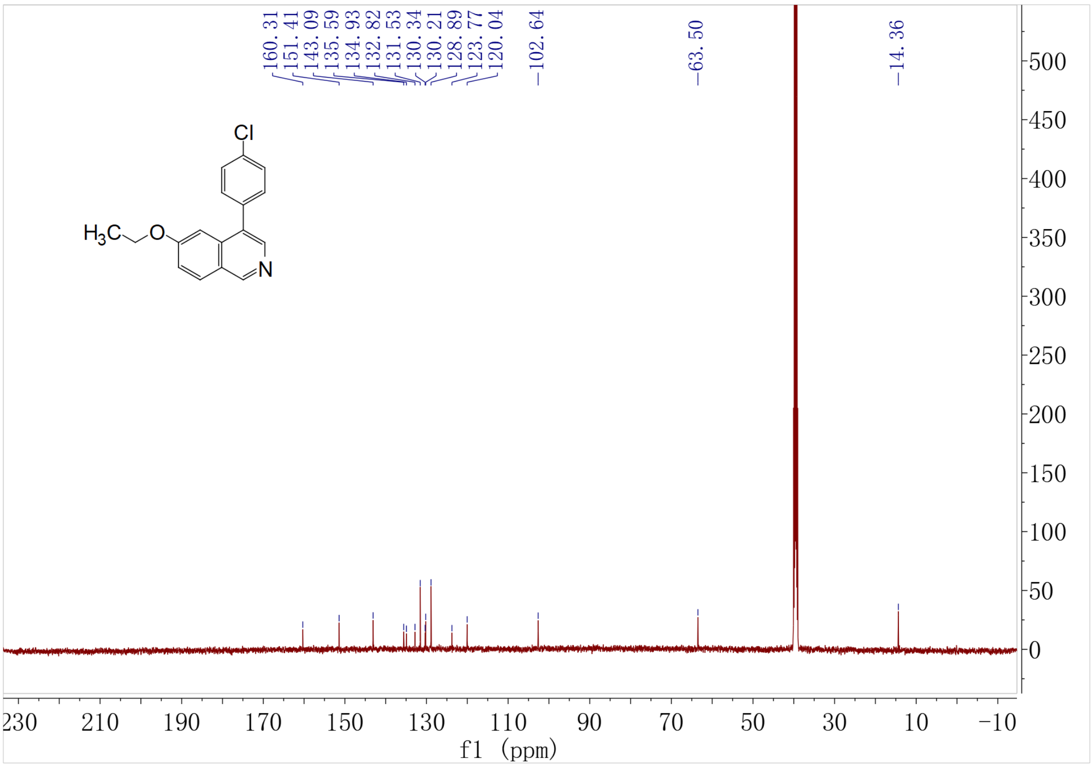

^{1}H NMR Spectrum of compound **39**

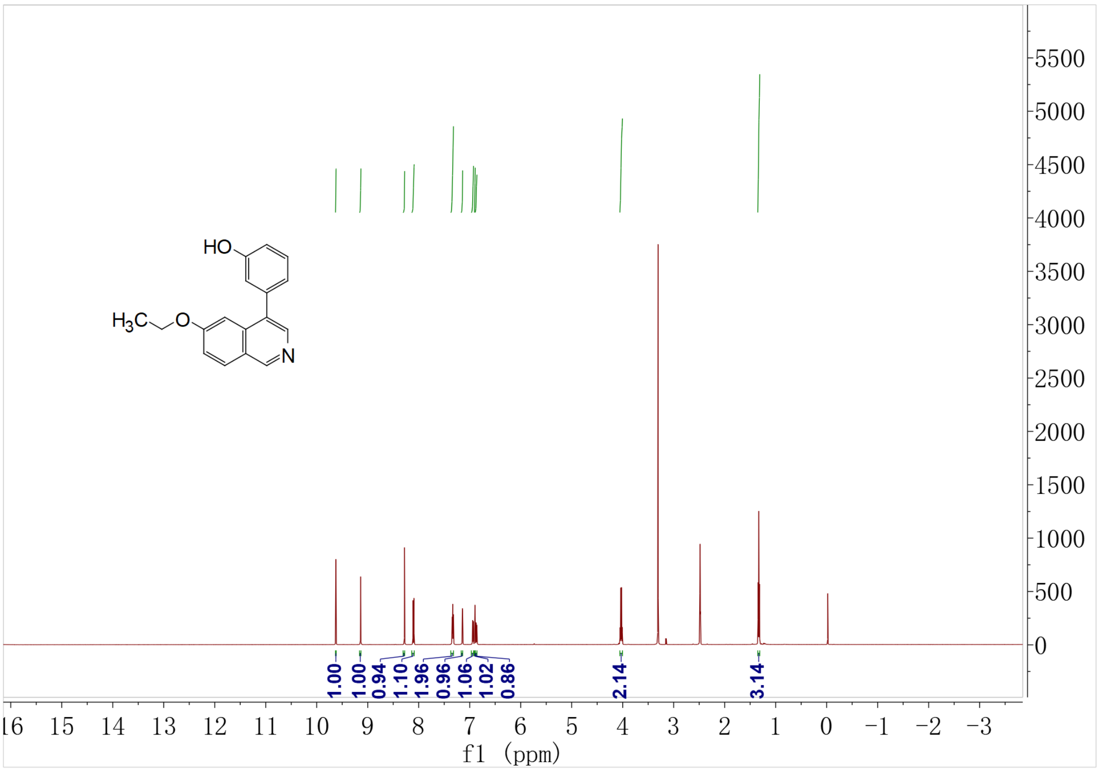

^{13}C NMR Spectrum of compound **39**

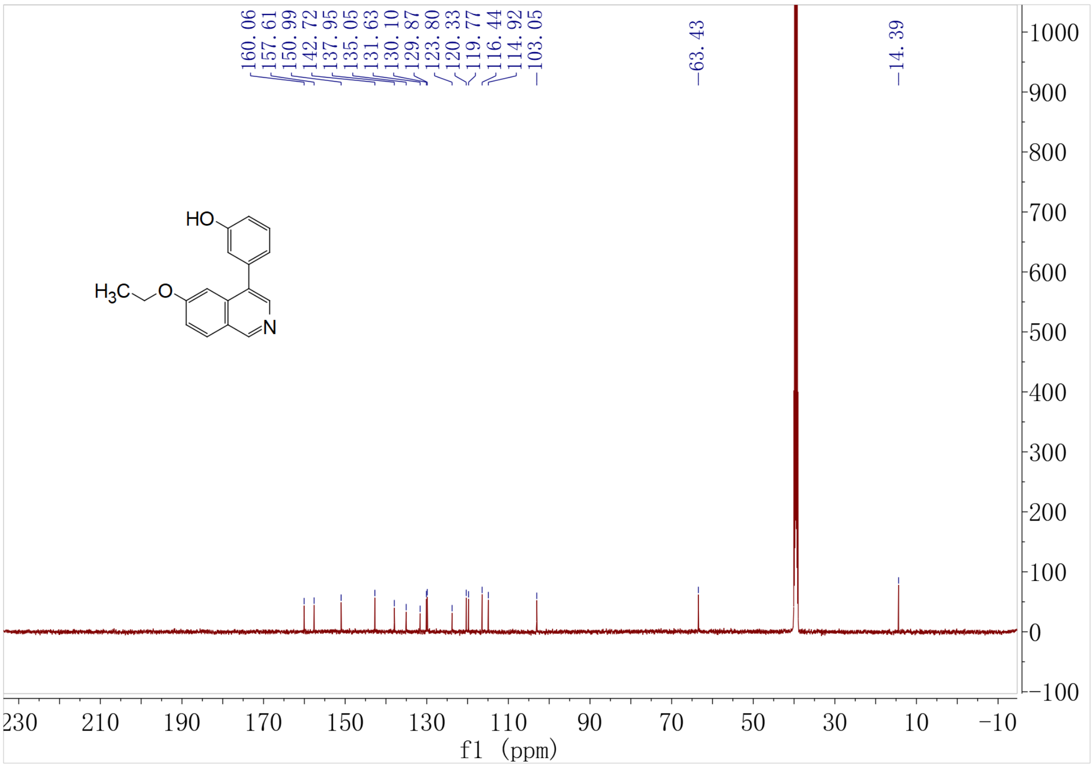

^{1}H NMR Spectrum of compound **40**

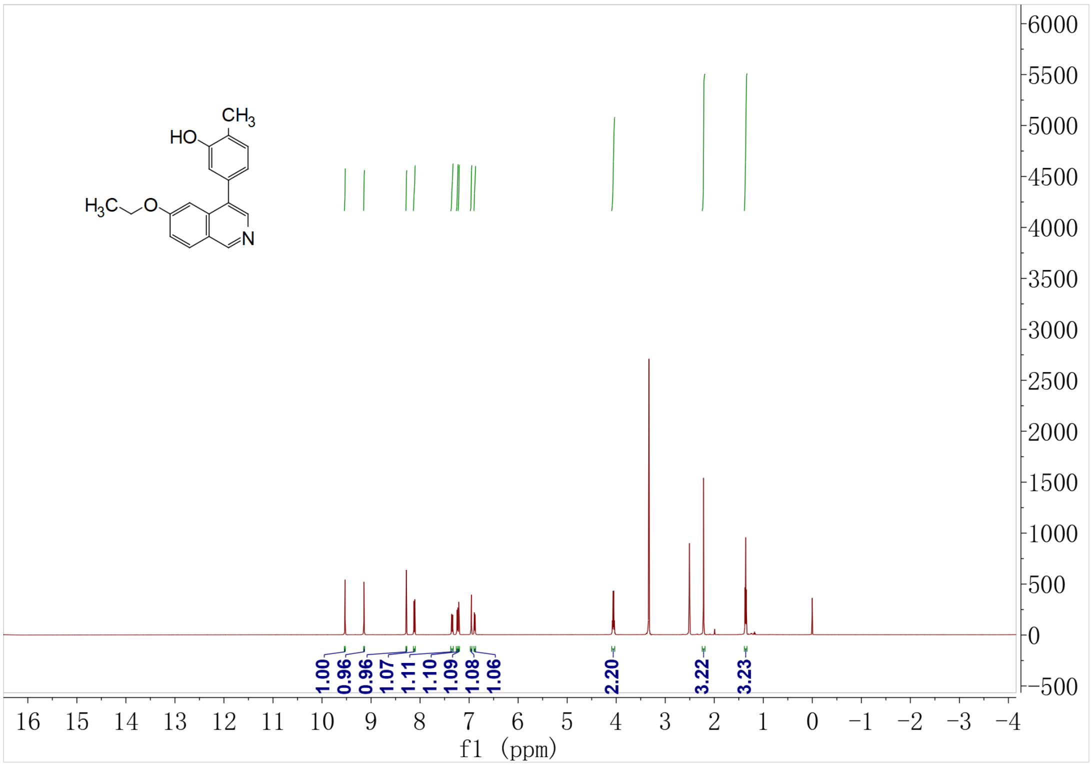

^{13}C NMR Spectrum of compound **40**

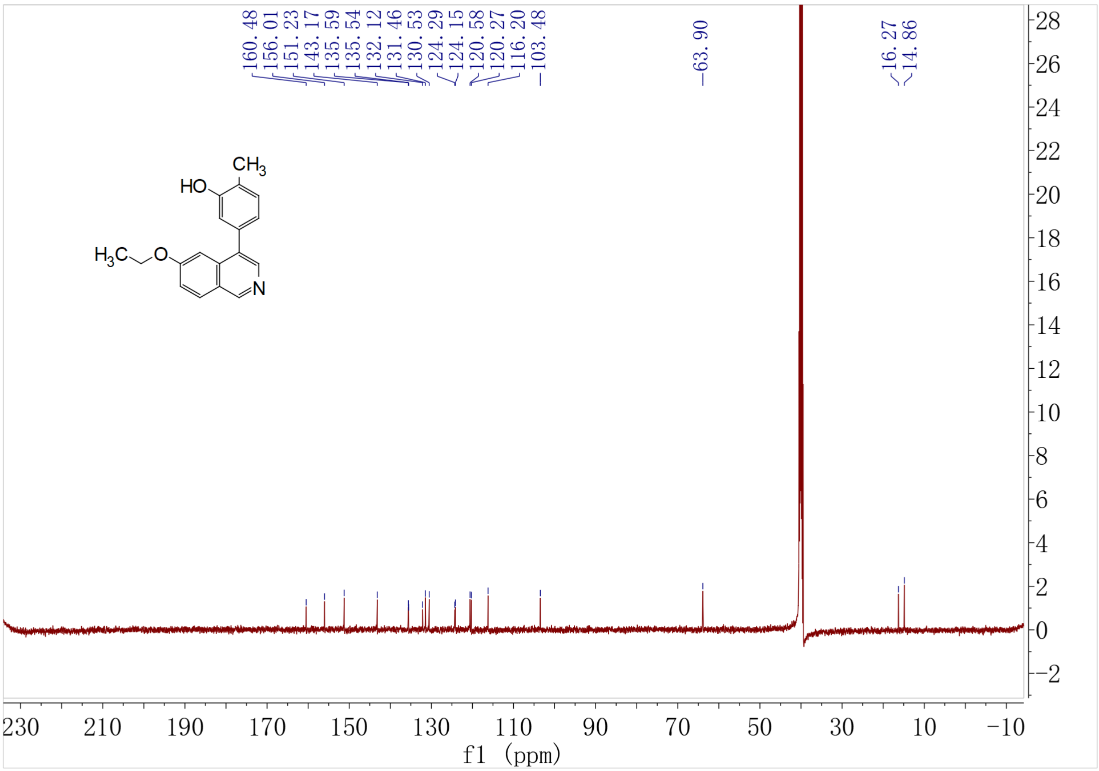

^{1}H NMR Spectrum of compound **45**

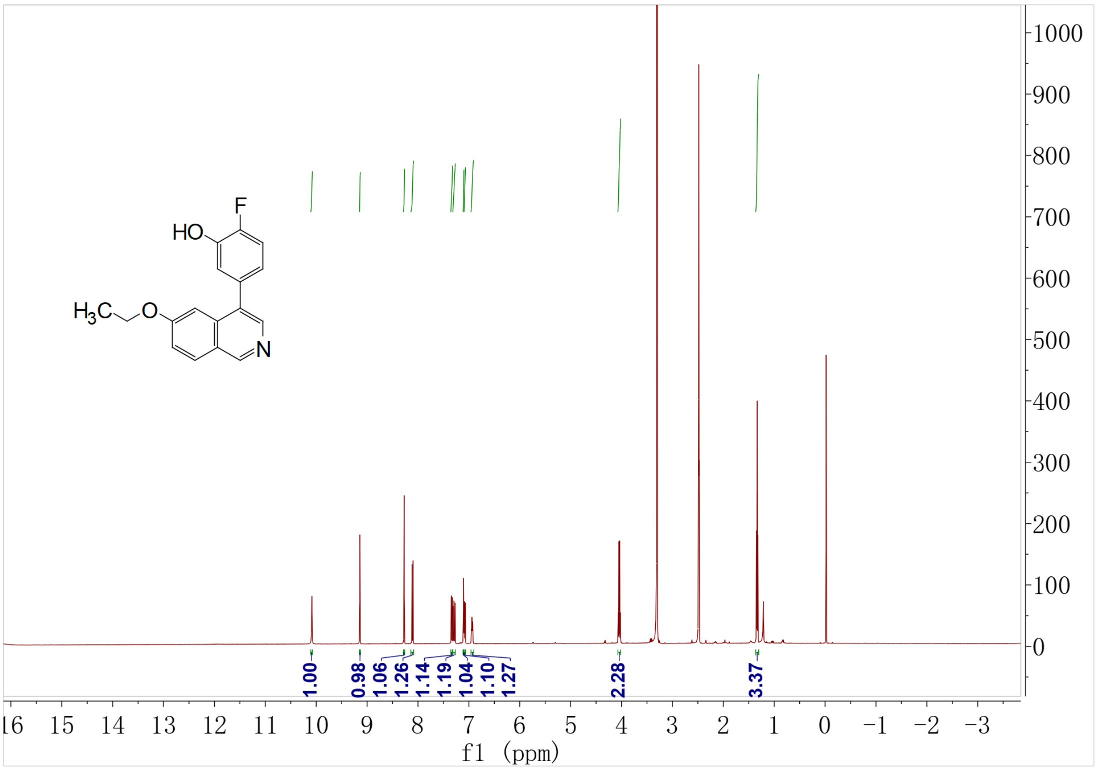

^{13}C NMR Spectrum of compound **45**

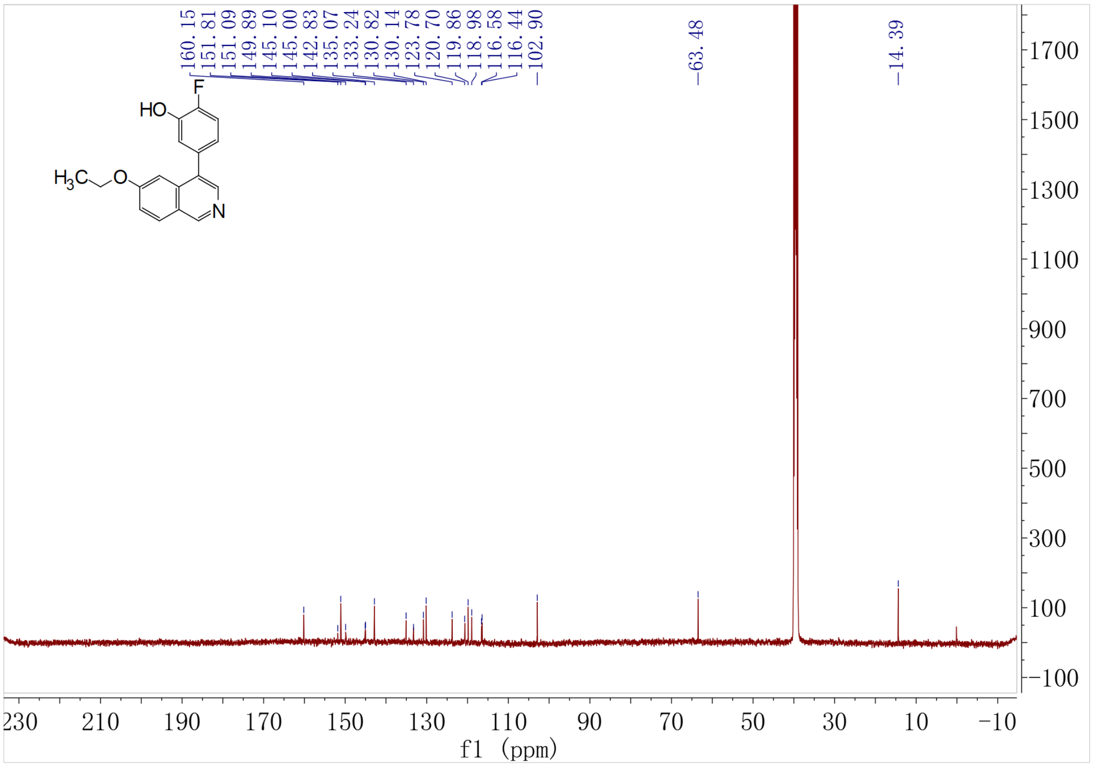

^{1}H NMR Spectrum of compound **46**

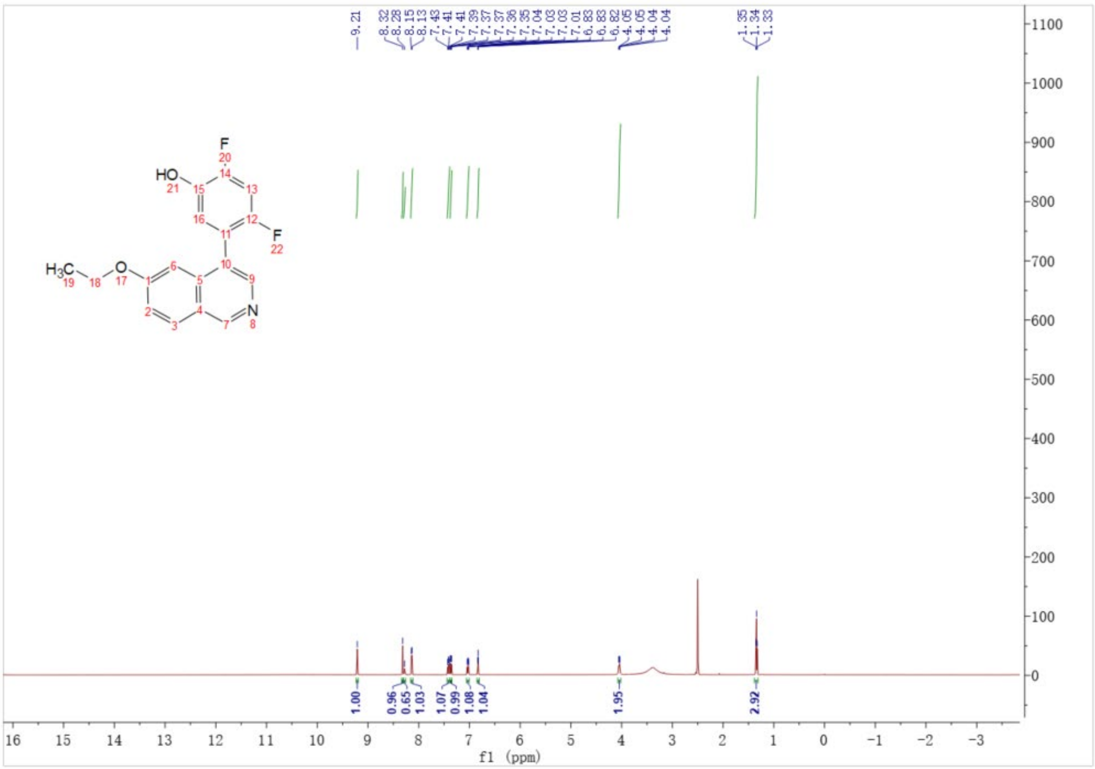

^{13}C NMR Spectrum of compound **46**

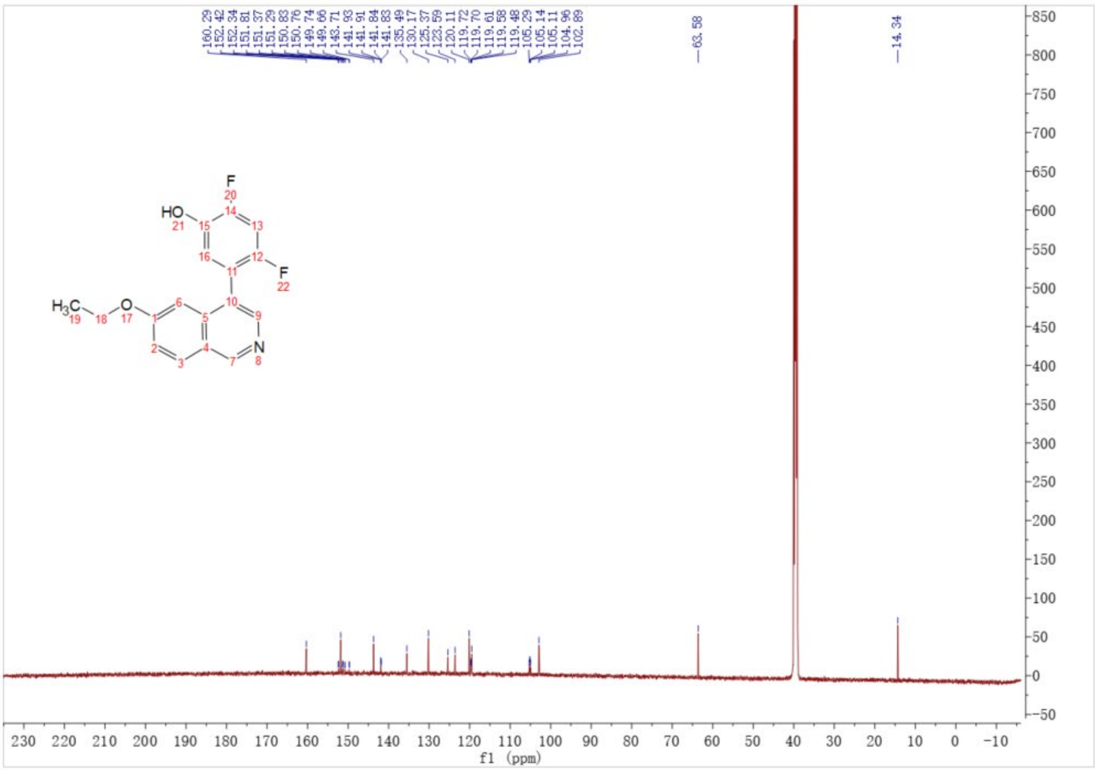

2. HPLC Spectrums

HPLC Spectrum of compound **1** (99.6% purity)

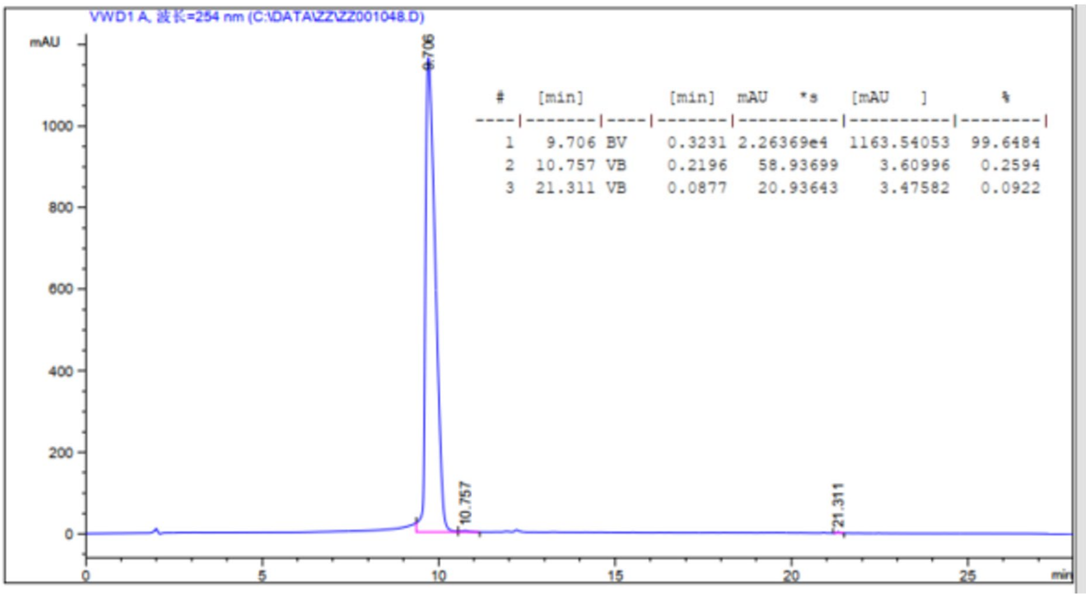

#	[min]		[min]	mAU *s	[mAU]	%
1	9.706	BV	0.3231	2.26369e4	1163.54053	99.6484
2	10.757	VB	0.2196	58.93699	3.60996	0.2594
3	21.311	VB	0.0877	20.93643	3.47582	0.0922

HPLC Spectrum of compound **20** (96.5% purity)

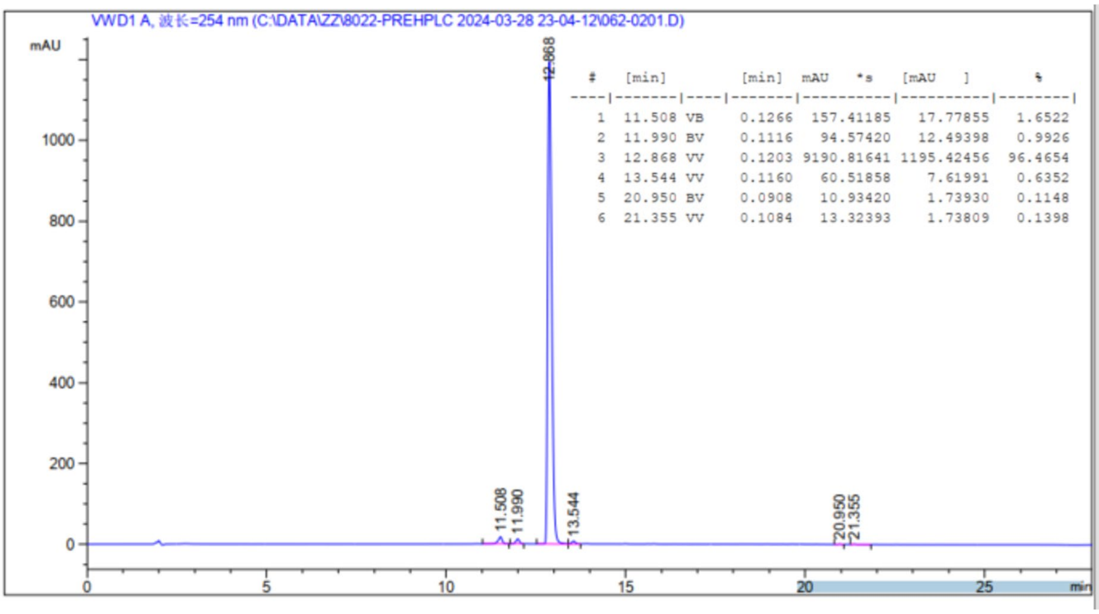

#	[min]		[min]	mAU *s	[mAU]	%
1	11.508	VB	0.1266	157.41185	17.77855	1.6522
2	11.990	BV	0.1116	94.57420	12.49398	0.9926
3	12.868	VV	0.1203	9190.81641	1195.42456	96.4654
4	13.544	VV	0.1160	60.51858	7.61991	0.6352
5	20.950	BV	0.0908	10.93420	1.73930	0.1148
6	21.355	VV	0.1084	13.32393	1.73809	0.1398

HPLC Spectrum of compound **23** (99.1% purity)

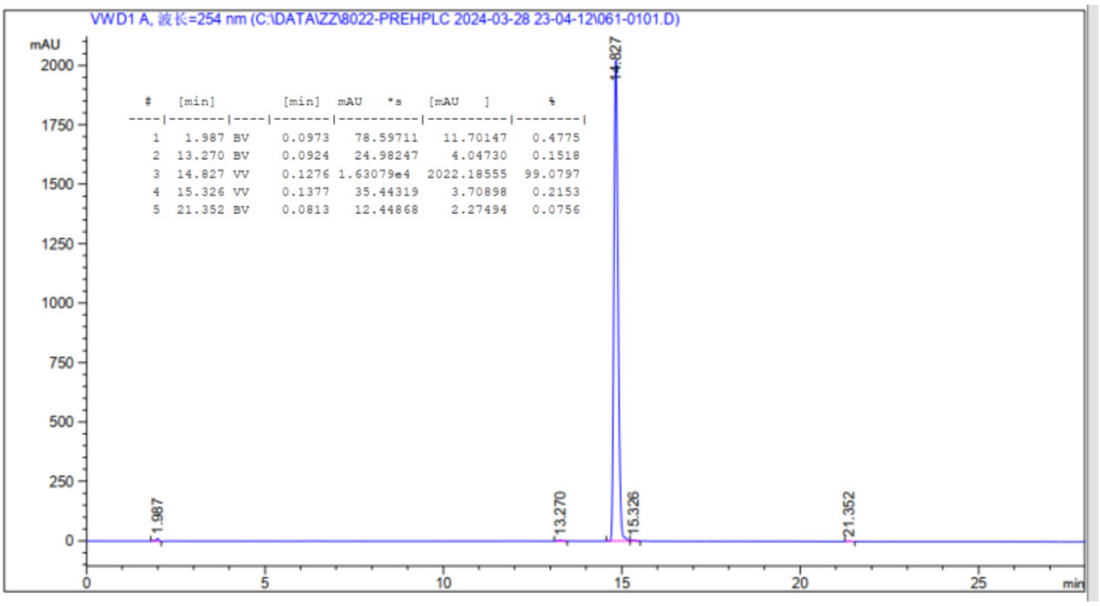

HPLC Spectrum of compound **46** (99.5% purity)

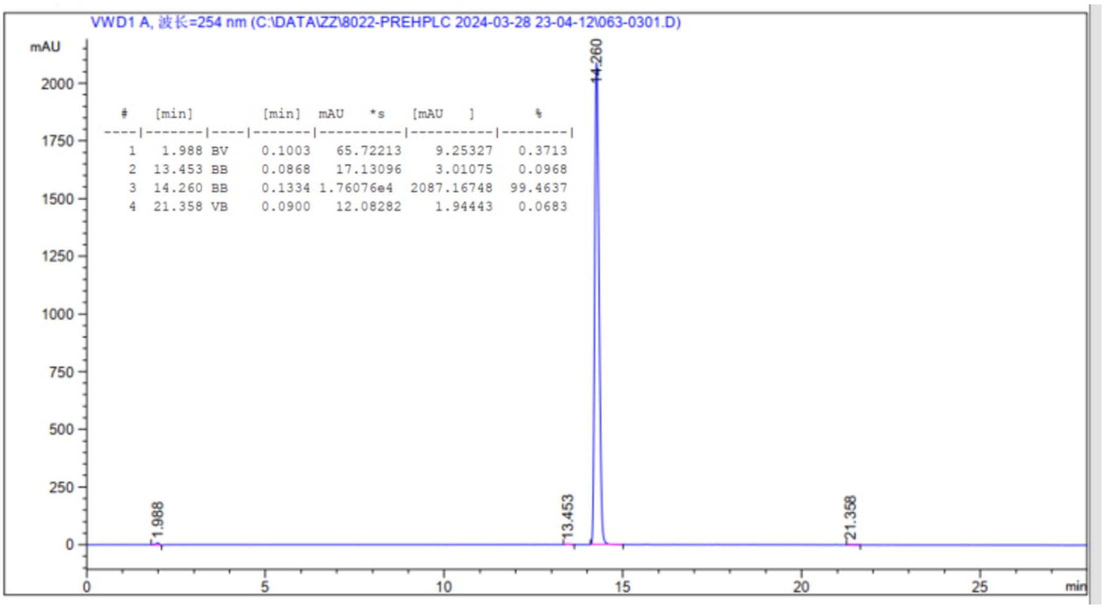

Supplement: Supplementary file 1 [file molecules-29-04503-s001.zip › molecules-3138300-supplementary.pdf]
